# Systematic Evaluation of Plasma and Urine Metabolites to Predict the Risk of Adverse Kidney-related Outcomes in Chronic Kidney Disease: The GCKD Study∗

**DOI:** 10.1016/j.xkme.2026.101409

**Published:** 2026-05-13

**Authors:** Elena Butz, Inga Steinbrenner, Ulla T. Schultheiss, Charlotte Behning, Harald Binder, Helena Hansmann, Wolfram Gronwald, Peter J. Oefner, Elke Schaeffner, Kai-Uwe Eckardt, Anna Köttgen, Peggy Sekula, GCKD Investigators

**Affiliations:** 1 Institute of Epidemiology and Prevention, Department of Data-Driven Medicine, Faculty of Medicine and Medical Centre – University of Freiburg, Freiburg, Germany; 2 Department of Medicine IV – Nephrology and Primary Care, Faculty of Medicine and Medical Centre – University of Freiburg, Freiburg, Germany; 3 Synlab MVZ Humangenetik Freiburg GmbH, Freiburg, Germany; 4 Institute for Medical Biometry, Informatics and Epidemiology, University Hospital Bonn, Bonn, Germany; 5 Institute of Medical Biometry and Statistics (IMBI), Department of Data-Driven Medicine, Faculty of Medicine and Medical Centre – University of Freiburg, Freiburg, Germany; 6 Centre for Integrative Biological Signalling Studies (CIBSS), University of Freiburg, Freiburg, Germany; 7 Department of Nephrology, Universitätsklinikum Regensburg, Regensburg, Germany; 8 Institute of Functional Genomics, University of Regensburg, Regensburg, Germany; 9 Institute of Public Health, Charité-Universitätsmedizin Berlin, Berlin, Germany; 10 Department of Nephrology and Medical Intensive Care, Charité-Universitätsmedizin Berlin, Berlin, Germany; 11 Department of Nephrology and Hypertension, Friedrich-Alexander-Universität Erlangen-Nürnberg, Erlangen, Germany; 12 Department of Epidemiology, Johns Hopkins Bloomberg School of Public Health, Baltimore, Maryland, USA

**Keywords:** Chronic kidney disease, CKD progression, kidney failure, prognosis, metabolites

## Abstract

**Rationale & Objective:**

Accurate risk prediction of adverse kidney-related outcomes in individuals with chronic kidney disease (CKD) is essential to guide personalized treatment. Plasma and urine metabolites may individually or jointly improve prediction beyond clinically established prognostic factors.

**Study Design:**

Prospective German CKD cohort study.

**Setting & Participants:**

5,217 individuals with predominantly CKD stage G3 at baseline and 6.5-year follow-up data (IQR, 6.5-6.5).

**Exposure(s) or Predictor(s):**

Baseline metabolite levels measured using untargeted mass spectrometry: plasma (N=5,144; 1,096 metabolites) and urine (N=5,088; 1,129 metabolites).

**Outcome(s):**

(i) Kidney failure (KF): kidney replacement therapy or death by untreated KF; (ii) composite kidney endpoint (CKE): KF, ≥ 40% estimated glomerular filtration rate (eGFR) decline, or eGFR < 15 mL/min/1.73 m^2^.

**Analytical Approach:**

Time-to-event analysis using subdistribution hazard models with component-wise boosting for metabolite selection. The predictive performance of metabolite models was compared to benchmark models, including established prognostic factors.

**Results:**

Several individual metabolites improved KF risk prediction beyond established prognostic factors (age, sex, eGFR, and urinary albumin-to-creatinine ratio). For example, adding plasma pseudouridine increased the area under the receiver operating characteristic curve (AUC) for KF at year 6 by 0.012 (95% CI, 0.005-0.018).

Multimetabolite models for KF (mean, 36 metabolites) showed good performance, declining for more distant time points: AUC values were ≥ 0.89 at year 2 and ≥ 0.85 at year 6. Some metabolites, such as plasma N2,N5-diacetylornithine and urine 1-palmitoyl-2-oleoyl-GPC (16:0/18:1), were selected more often than others. Overall, multimetabolite models demonstrated modest, partially significant improvements over clinical models, and were comparable to other suggested prognostic models of KF. Results for the CKE were similar.

**Limitations:**

Single-point, semiquantitative metabolite measurements.

**Conclusions:**

While certain metabolites improved the prediction of adverse kidney-related outcomes, added value was limited. However, prognostic metabolites may reflect relevant CKD-related metabolic pathways. Further research is warranted to refine prognostic models and explore the biological relevance of identified metabolites.

## Plain-Language Summary

This study examined whether measuring small molecules (metabolites) in blood and urine can improve the prediction of kidney failure risk in more than 5,000 people with chronic kidney disease over 6 years. Some metabolites slightly improved risk prediction beyond common clinical factors such as sex, age, kidney function (the kidney’s ability to remove waste and toxins), and kidney impairment (when protein leaks into the urine). We therefore evaluated whether models combining multiple metabolites provided additional benefits. As a result, these multimetabolite prognostic models provided reliable and clinically meaningful risk estimates of kidney failure, but their improvement over existing clinical models was small. However, metabolites selected into multimetabolite models may reflect important biological processes and could help guide future research.

## Introduction

Disease courses of individuals with chronic kidney disease (CKD) are highly variable.^1^^,^^2^ It is thus clinically important to identify individuals at higher risk for adverse outcomes such as kidney failure (KF) to enable earlier and more personalized interventions.^3^, ^4^, ^5^, ^6^

Several prognostic models for KF have been developed.^7^ The most widely used model is the kidney failure risk equation (KFRE), which includes age, sex, estimated glomerular filtration rate (eGFR), and urinary albumin-to-creatinine ratio (UACR).^8^ The KFRE has been validated in diverse settings, but performance varies across studies.^7^^,^^9^, ^10^, ^11^, ^12^, ^13^ Although discrimination is often adequate, substantial unexplained variance remains. For example, Tangri et al^9^ reported 5-year C statistics ranging from 0.77-0.96. Calibration is also inconsistent, with notable underestimation or overestimation in some populations, including those with advanced CKD.^11^ These discrepancies may reflect differences in study settings or methods, as well as the inherent difficulty of predicting a multifactorial outcome. Attempts to improve KF risk prediction with additional clinical parameters have shown limited success.^6^^,^^7^^,^^10^^,^^14^^,^^15^

Metabolites are promising prognostic markers in nephrology because they reflect (patho-)physiological processes and are often cleared by the kidneys.^16^^,^^17^ Several studies, including our own work within the German CKD (GCKD) study,^18^ have examined associations between metabolite levels and adverse CKD outcomes.^17^^,^^19^, ^20^, ^21^ Recently, we showed for a limited set of metabolites that some can improve the risk prediction of adverse kidney outcomes beyond the four KFRE variables.^19^^,^^21^

Building on these findings, we systematically explored the value of all detected metabolites to identify new prognostic factors explaining remaining KF variance. Beyond evaluating individual metabolites, we used a feature selection algorithm to develop multimetabolite models, combining predictive values of single metabolites (Fig S1). Established prognostic factors like the KFRE variables were considered. We then compared the predictive performance of these models to benchmark models (ie, the same models without metabolites) to determine the metabolites’ added value in predicting risks of adverse kidney-related outcomes.^8^^,^^22^ We also examined whether plasma or urine metabolites alone yielded better-performing models and whether measured metabolites not fully identified carry predictive information.

## Methods

### Study population

The GCKD study is an ongoing multicenter prospective cohort study (DRKS 00003971) that enrolled 5,217 participants with CKD between 2010 and 2012.^18^ Major inclusion criteria were (a) an eGFR between 30 and 60 mL/min/1.73 m^2^, or (b) overt albuminuria or proteinuria with an eGFR > 60 mL/min/1.73 m^2^. All participants had been referred to nephrologists and provided informed consent. The local ethics committees of the participating centers approved the study (Item S1).

### Baseline data and measurements

At baseline, participants were systematically assessed by trained study personnel to collect demographic and clinical data. Biospecimens, including plasma and spot urine, were obtained and transported frozen to a central biobank, following standardized protocols.^23^ Some laboratory parameters were measured immediately, while other samples were stored for future analysis. Details of the study cohort have been published previously.^24^ Variables used in this analysis are listed in Item S2. eGFR was calculated using the CKD-EPI creatinine-based equation,^25^ consistent with our prior work.^21^

Baseline urine and plasma samples were analyzed by Metabolon Inc using untargeted liquid chromatography-mass spectrometry. Urine samples were processed in 3 batches between 2016 and 2017 and plasma samples in a single batch in 2020. Details on sample preparation, mass spectrometry procedures, and metabolite identification have been described previously,^21^^,^^26^ with a summary provided in Item S3. Processing included repetition of urine metabolite identification at the time plasma samples were processed and normalization of levels to account for interday instrument variation. Named metabolites were assigned to superpathways (eg, amino acids and xenobiotics). Unless marked with an asterisk, named metabolites met the highest confidence level (level 1) per Metabolomics Standards Initiative criteria.^27^^,^^28^ Some metabolites were partially characterized or unnamed, lacking a defined structural identity.

Released semiquantitative measurements of metabolites underwent further quality control and preprocessing (Item S4). This included normalization of urine measurements to harmonize differences in dilution using the probabilistic quotient method^29^ and log_2_-transformation of all measurements. Missing values were imputed using the k-nearest neighbor (*knn*) algorithm,^30^ enabling the application of feature selection methods for developing multimetabolite models. Only metabolites with sufficient measurements were imputed: > 95% for xenobiotics and > 50% for nonxenobiotics. As a result, 704 metabolites (609 named) were excluded because of high proportions of missing data (Table S1). Quality assessment of the imputed data did not indicate any issues (see Item S5 for details).

The final dataset included measurements of 1,096 plasma metabolites from 5,144 individuals and 1,129 urine metabolites from 5,088 individuals (Fig S1; Tables S2 and S3). Of the included metabolites, 25% in plasma and 38% in urine were unnamed. A total of 583 metabolites (417 named) were detected in both plasma and urine. Paired plasma and urine measurements were available for 5,023 participants.

### Prospective data and outcomes

Events were identified using hospital discharge summaries, nephrologist outpatient reports, and death certificates, and were adjudicated by a medical expert team using a standardized event catalog.^31^ In line with our previous work,^21^ follow-up data up to 6.5 years after study enrollment were included in the analysis (data freeze: March 31, 2021).

The primary outcome was KF, defined as the initiation of kidney replacement therapy (maintenance dialysis or kidney transplantation) or death by untreated KF. As a secondary endpoint, we analyzed a composite kidney endpoint (CKE), which included KF, a ≥ 40% decline in eGFR, and an eGFR < 15 mL/min/1.73 m^2^. eGFR-based components of CKE were determined from longitudinal creatinine measurements using linear mixed models.^32^ On average, nine creatinine values (IQR, 7-12) per participant were available.

### Statistical analysis

All analyses were performed using R (version 4.0.5 or 4.3.3, R Core Team (2020) – R: a language and environment for statistical computing. R Foundation for Statistical Computing, http://www.R-project.org/). This project involved 2 primary steps (Fig S1): First, evaluating the ability of individual metabolites to predict risk of KF and CKE, and second, developing multimetabolite models and evaluating their predictive performance (Item S6).

To estimate the probability of the event until year 6, cumulative incidence functions were estimated using the R package *etm*.^33^

(i) *Regression models*: Subdistribution hazard models were chosen to predict the risk of outcomes, since they account for competing risks such as death from causes other than untreated KF.^34^^,^^35^ Competing risks need to be acknowledged because they reduce the risk for the event of interest, especially if the proportion of competing events is non-negligible (GCKD cohort: 500 KF events vs 551 competing death events).^36^ Outcomes were defined as the time from study entry to either the event of interest or a competing event, whichever occurred first. Individuals who experienced neither nor were lost to follow-up were censored at their last known time alive within a period of a maximum of 6.5 years.

To assess the predictive value of metabolites alone and in addition to known prognostic factors, we evaluated three model configurations (Item S6):

(1) A model without any prognostic factors (= null model; Bench) versus a model with metabolites only (MET).
(2) A model with KFRE variables (age, sex, eGFR, and ln-transformed UACR value; Bench+)^8^ versus a model that additionally included metabolite(s) (MET+).
(3) A model with an extended set of 14 clinical prognostic factors (Bench++) versus a model that additionally included metabolite(s) (MET++).
  The extended set of clinical prognostic factors corresponds to the comprehensive model used in our previous project^21^ and includes variables selected based on a thorough literature review and biological rationale (Item S2, Status: August 2021).
(ii) *Handling of missing observations*: After imputation, metabolite data were complete for all individuals with any available plasma or urine metabolites (Item S4). Clinical prognostic factors had minimal missingness (0%-2%; Item S2).

For single metabolite models (step 1), we used 2 GCKD cohort subsets restricted to individuals with complete KFRE data and the relevant plasma (N=5,047) or urine (N=5,025) metabolite measurements (Fig S1). For multimetabolite models (step 2), we included only individuals with complete data for all 14 prognostic factors and both plasma and urine metabolites (N=4,886; Item S2). Using the same sample in step 2 ensured comparability of predictive performances.

(iii) *Multimetabolite model construction*: For each configuration (Item S6), a multimetabolite model was derived. To select metabolites and to fit multimetabolite models, the CoxBoost algorithm, a component-wise boosting algorithm for subdistribution hazard models, was employed (v1.5; https://github.com/binderh/CoxBoost).^37^ CoxBoost builds a prognostic model stepwise by updating one variable at a time, selecting at each step the metabolite that most improves the prediction of the time-to-event outcome while accounting for competing events. By using a prespecified number of boosting steps using 10-fold cross-validation, this approach produces a parsimonious model that retains the most informative metabolites while avoiding overfitting. It also allows prespecified variables, such as clinical covariates, to be included throughout the selection process. Only metabolites were subjected to feature selection, while clinical prognostic factors were kept mandatory if present in the model. Detailed descriptions of the selected parameters are provided in Item S7. Plasma creatinine level was measured using Metabolon, which was retained as a positive control, allowing CoxBoost to select it among the candidate metabolites.
(iv) *Assessment of predictive performance*: To comprehensively assess the predictive performance of each model, we used the R package *riskRegression* (version 2023.01.19; https://github.com/tagteam/riskRegression; Item S8).^38^^,^^39^ *CoxBoost* objects were made compatible with *riskRegression* using a wrapper function (Item S9).

Discrimination between individuals with and without the event of interest at 2, 4, 5, and 6 years after study entry was assessed using time-dependent area under the receiver operating characteristic curve (AUC) values^39^^,^^40^ and the prediction error (Brier score).^41^ We assessed the added predictive value based on differences in AUC (ΔAUC) value and Brier score (ΔBrier) between each multimetabolite model and its benchmark (Item S6). In addition, we provided estimates of the net reclassification index^42^ and the integrated discrimination improvement^43^ based on the package *survIDINRI*.^44^ We internally validated our results using bootstrap cross-validation. Bootstrap samples of the same size as the original cohort were drawn with replacement (N=100), models were developed in each resampled dataset, and their performance was evaluated in the corresponding out-of-bootstrap observations.^22^ Unless indicated, all reported AUC values and Brier scores refer to these bootstrap cross-validated estimates.

For further comparison, we applied other published proposals of models predicting KF risk to our analysis data.^14^^,^^15^^,^^45^ Notably, the model proposed by Zacharias et al^45^ was also developed using GCKD study data, albeit with a shorter follow-up period of 4 years.

The reporting guideline Reporting Recommendations for Tumor Marker Prognostic Studies for prognostic factor studies was considered when drafting the manuscript.^46^

## Results

### Study population and metabolite data

In the main analysis cohort used to derive multimetabolite models (N=4,886), the proportion of males was 60%, the mean age was 60 years, the mean eGFR was 49.5 mL/min/1.73 m^2^ (±SD 18.3), and the median UACR value was 50.4 (IQR, 9.5-385) mg/g (Table 1). Prevalent morbidities at baseline included hypertension (96%), diabetes (35%), coronary heart disease (20%), and stroke (10%).

**Table 1:** Sample characteristics for the GCKD cohort and the main analysis set

Variable	GCKD Cohort	Cohort With Plasma and Urine Metabolites^a^
Sample size, N	5,217	4,886
Sex, male	60 (3,132)	60 (2,947)
Age (y)	60.1 (12.0)	60.0 (12.0)
eGFR (mL/min/1.73m^2^)	49.4 (18.3)	49.5 (18.3)
UACR (mg/g)	50.9 (9.7-392)	50.4 (9.5-385)
Smoking	41 (2,131)	41 (1,987)
nonsmoker		
ex-smoker	43 (2,242)	43 (2,122)
current smoker	16 (828)	16 (777)
Body mass index (kg/m^2^)	29.82 (5.97)	29.75 (5.92)
Blood pressure, systolic (mmHg)	139.49 (20.36)	139.3 (20.27)
Serum albumin level (g/L)	38.33 (4.29)	38.34 (4.28)
Serum C-reactive protein level (mg/L)	2.29 (1.03-5.03)	2.24 (1.01-4.88)
Serum cholesterol level (mg/dL)	211.18 (52.84)	211.29 (52.63)
Diabetes	36 (1,868)	35 (1,717)
Coronary heart disease	20 (1,039)	20 (971)
Stroke	10 (510)	10 (470)
Hypertension	96 (5,021)	96 (4,701)
Blood pressure-lowering drug use, any	94 (4,901)	94 (4,589)
Blood pressure-lowering drug use, loop diuretics	39 (2,014)	38 (1,861)
Overall follow-up time (in d)	2,374 (0-2,374)	2,374 (0-2374)
Outcome: kidney failure	10 (500)	10 (465)

Number of missing values: eGFR values 55, UACR values 90, smoking 16, BMI values 54, blood pressure measurements 34, serum albumin levels 57, serum C-reactive protein levels 56, serum cholesterol levels 62, coronary heart disease 2, stroke 2, hypertension 3.

For continuous variables, mean (±SD) is provided, median (IQR) for UACR, and median (range) for follow-up time.

Abbreviations: eGFR, estimated glomerular filtration rate; GCKD, German chronic kidney disease; UACR, urinary albumin-to-creatinine ratio.

a In the main analysis (Fig S1), the GCKD cohort was restricted to participants with plasma and urine measurements who also have complete information on the extended list of prognostic factors, which are all listed in this table (see also Item S2).

Over a median follow-up of 6.5 (IQR, 6.5-6.5) years, 465 (10%) incident KF events were observed, resulting in a probability for the event at year 6 of 0.10 (95% CI, 0.09-0.11; Fig S2). For the secondary endpoint CKE, which was experienced by 1,010 (21%) participants, the probability for the event at year 6 was 0.22 (95% CI, 0.21-0.23; Fig S2).

Overall, baseline characteristics of this and other analyzed subcohorts were comparable with those of the full GCKD study population (Table S4).

### Predictive value of individual metabolites

The evaluation of the predictive value of single metabolites revealed that several metabolites showed strong discriminatory power when used alone (MET model). Plasma pseudouridine achieved the highest observed AUC value for predicting KF risk at year 6 (AUC value, 0.80; 95% CI, 0.77-0.83; Table S5).

The predictive performance of individual metabolites, as measured using AUC values, generally declined for more distant time points. For instance, the AUC value of plasma pseudouridine for KF risk prediction decreased from 0.88 at year 2 (95% CI, 0.84-0.91) to 0.81 at year 5 (95% CI, 0.78-0.84).

Adding individual metabolites to the Bench+ model rarely resulted in substantial improvements in AUC value and Brier score, suggesting limited added predictive value in most cases (Fig S3). Nonetheless, 59 plasma metabolites and 13 urine metabolites statistically significantly improved the prediction of KF risk at year 6 (Fig S3; Tables S5 and S6). For example, inclusion of plasma pseudouridine improved the AUC value at year 6 to 0.86 (95% CI, 0.84-0.88), representing a gain of 0.012 (95% CI, 0.005-0.018) over the Bench+ model. Similar results were observed for CKE (Fig S4; Tables S7 and S8).

### Composition of multimetabolite models

Multimetabolite models to predict the risk of KF were derived for a total of 6 distinct settings: 3 using only named metabolites, and 3 including both named and unnamed metabolites. In each case, the optimal number of boosting steps was determined before the selection started (Table S9). For instance, to develop a MET model predicting KF risk using only named metabolites, 100 boosting steps were determined and yielded 27 selected metabolites. The coefficient trajectories of selected metabolites across boosting steps are illustrated in Fig S5, where the coefficient of a single metabolite is updated at each step.

Across the 6 models, the number of selected metabolites to predict KF risk (mean, 36; range, 22-67) increased with the number of boosting steps (mean, 122; range, 52-229; Pearson correlation coefficient r = 0.98). At the same time, the number of selected metabolites decreased as more clinical variables were mandatorily included. This pattern suggests that some metabolites may be considered as potential proxies for clinical variables, while others provide additional predictive value even in the presence of clinical information. Models predicting the risk of the secondary outcome CKE generally required more boosting steps and selected more metabolites than those predicting KF risk.

**Figure 1:**
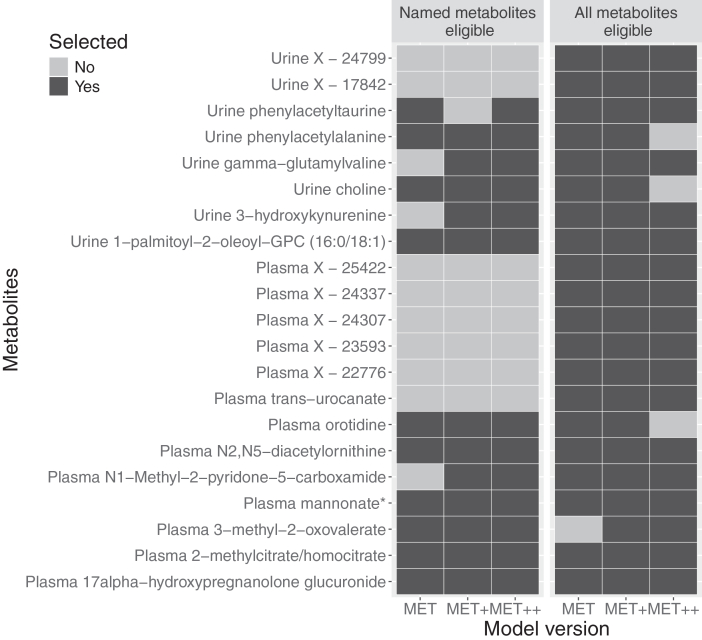
Overview of metabolites selected in all models predicting kidney failure risk with either only named or all metabolites eligible. Multimetabolite models were derived under 3 different settings (Item S6), with either only named metabolites eligible or all metabolites eligible. This heatmap includes metabolites that were chosen in all 3 settings of either set of eligible metabolites. Abbreviations: MET, multi-metabolite model without clinical information; MET+, multi-metabolite model including KFRE variables; MET++, multi-metabolite model including extended set of prognostic factors (see Item S2 for details).

In the 3 KF prediction settings limited to named metabolites, 42 distinct metabolites were selected at least once (Table S10), with 9 selected in all 3 settings (Fig 1). As expected, plasma creatinine level was selected only in MET models predicting KF risk, which did not include eGFR (Table S10). When unnamed metabolites were included, the total number of metabolites selected at least once increased to 95, although not all previously selected named metabolites were retained. Five of the 9 metabolites consistently selected in the named-only settings were again selected in all 3 settings with all metabolites available, alongside 7 unnamed metabolites, highlighting the potential added relevance of unnamed metabolites (Fig 1; Table S10).

For CKE, 98 named metabolites were included in the 3 respective settings, with 24 of them selected each time (Fig S6). When including unnamed metabolites, 16 out of 24 metabolites were also selected each time.

The whole selection process seemed to be fairly stable when repeated 100 times (Item S10; Table S11).

### Predictive value of multimetabolite models

At 6 years, all 6 multimetabolite models for KF risk prediction slightly outperformed their benchmarks in AUC value, Brier score, net reclassification index score, and integrated discrimination improvement score (Figs S7-S9). Bootstrap cross-validation attenuated performance estimates, but all models still discriminated KF risk effectively, with cross-validated AUC values≥ 0.86 (Table 2). Several models significantly improved both the AUC value and Brier score compared with the benchmark values.

**Table 2:** Bootstrap cross-validated values of the area under the ROC curve and Brier score at year 6 for the prediction of kidney failure risk

Configuration	Model Without Clinical Information	Model Including four KFRE Variables	Model Including Extended Set of Prognostic Factors
Comparison	MET	MET+ vs Bench+	MET++ vs Bench++
AUC value (95%CI)			
Benchmark model (w/o metabolites)	*-*	0.84 (0.82-0.87)	0.86 (0.83-0.88)
Multimetabolite model based on named metabolites	0.86 (0.84-0.88)	0.86 (0.83-0.89)	0.87 (0.84-0.89)
ΔAUC value (95% CI)	-	**0.019 (0.009-0.029)**	0.010 (0.000-0.021)
Multimetabolite model based on all metabolites	0.86 (0.84-0.88)	0.87 (0.84-0.89)	0.87 (0.84-0.89)
ΔAUC value (95% CI)	-	**0.022 (0.011-0.33**)	0.011 (0.000-0.023)
Brier score (95% CI)			
Benchmark model (w/o metabolites)	-	0.065 (0.058-0.073)	0.068 (0.059-0.076)
Multimetabolite model based on named metabolites	0.065 (0.058-0.072)	0.064 (0.056-0.072)	0.065 (0.056-0.073)
ΔBrier score (95% CI)	-	−0.002 (−0.004 to 0.000)	**−0.003 (−0.006 to 0.000)**
Multimetabolite model based on all metabolites	0.066 (0.058-0.072)	0.064 (0.056-0.072)	0.065 (0.056-0.073)
ΔBrier (95% CI)	-	−0.001 (−0.004 to 0.001)	−0.003 (−0.006 to 0.000)

Significant contrasts (ΔAUC value and ΔBrier score) are bold.

Abbreviations: Δ, contrast of AUC or Brier score between multimetabolite model and respective benchmark model; 95% CI, 95% confidence interval; AUC, area under the receiver operating characteristic curve; Bench+/Bench++, model containing respective set of clinical information without metabolites; KFRE, kidney failure risk equation which contains age, sex, estimated glomerular filtration rate and urinary albumin-to-creatinine ratio; MET, multimetabolite model without clinical information; MET+, multimetabolite model including KFRE variables; MET++, multimetabolite model including extended set of prognostic factors (see Item S2 for details); NA, not applicable; w/o, without.

**Figure 2:**
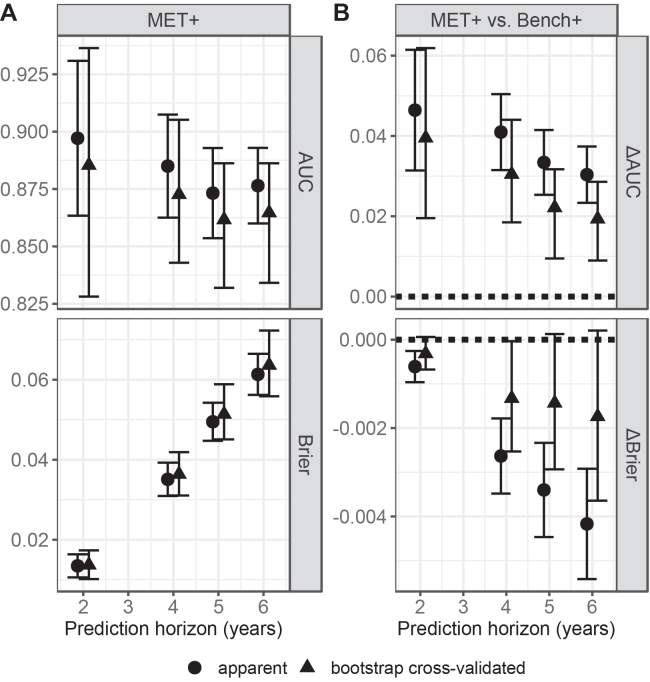
Performance of the model with named metabolites in absolute terms as well as in comparison to its benchmark model. The MET+ model with named plasma and urine metabolites performs well, but its performance measures decline for more distant time points (A). The model reaches a higher AUC value than its benchmark model but does not significantly improve prediction in terms of cross-validated Brier scores (B). Abbreviations: AUC, area under the receiver operating characteristic curve; ΔAUC value, difference in AUC values; Bench+, model containing only KFRE variables and no metabolites; ΔBrier, difference in Brier score; KFRE, kidney failure risk equation, which contains age, sex, estimated glomerular filtration rate, and urinary albumin-to-creatinine ratio; MET+, multimetabolite model including KFRE variables (see Item S2 for details).

The performance measures of both benchmark and multimetabolite models decreased at more distant time points (Tables S12 and S13), as illustrated for named MET+ models in Fig 2. Adding selected metabolites to the Bench+ model significantly increased AUC values. The greatest improvement was obtained by adding plasma and urine metabolites (including unnamed) with a ΔAUC value = 0.046 (95% CI, 0.017-0.076) observed at year 2, 0.026 (95% CI, 0.012-0.037) at year 5, and 0.022 (95% CI, 0.011-0.033) at year 6 (Table S14). In contrast, when metabolites were added to the Bench++ model, improvements were smaller and no longer statistically significant at years 5 and 6. Reductions in prediction error (Brier score) were generally not statistically significant.

When we applied previously published models^14^^,^^15^^,^^45^ to our data, their performance in predicting risk of KF (AUC value range, 0.84-0.88) was comparable with our multimetabolite models, likewise providing small gains over the model with the four KFRE variables (Table S15). Finally, when restricting the selection for multimetabolite models to either plasma or to urine metabolites, both yielded similar predictive performances, with no evidence that one matrix outperformed the other (Fig S10; Tables S12-S14).

For CKE, multimetabolites and benchmark models showed reduced performance, whereas the conclusions were similar to those for models predicting KF risk (Tables S16-S18).

## Discussion

This exploratory study demonstrates that both single metabolites and multimetabolite models are capable of predicting the risk of adverse kidney-related outcomes in individuals with established CKD. Although we initially expected that combining metabolites into multimetabolite models would enhance predictive performance, the observed improvements—while statistically significant in some cases—did not reach a magnitude that could be considered clinically meaningful. This may be because the variables already included in the KFRE yielded an AUC value of 0.84 for 6-year KF risk prediction in our study, leaving limited room for further improvement. In addition, some metabolites may capture the same predictive information as clinical variables.

Across various settings, several metabolites were selected in all models. Since the development of prognostic models is aimed solely at optimizing risk prediction, the selection of metabolites does not consider their causal relationship with the outcome or any potential confounders.^47^ Still, the selected metabolites may reflect information on biological mechanisms. For example, plasma pseudouridine alone achieved an AUC value of 0.80 (95% CI, 0.77-0.83) in predicting KF risk (Table S5). Pseudouridine has been previously linked to kidney function and CKD by several research groups (for details, see Table S7 of Steinbrenner et al^21^). It is a posttranslationally modified nucleoside found in RNA, continuously produced and freely filtered by the kidneys. However, its net reabsorption limits its use as a filtration marker.^48^^,^^49^ Its connection to kidney function explains its selection in multimetabolite models predicting KF risk only in the absence of eGFR. A similar situation is observed with plasma creatinine level.

Plasma N2,N5-diacetylornithine, which was always selected for KF (Fig 1), was not identified as associated with KF or CKE in our previous project.^21^ However, other studies have reported associations with eGFR, proteinuria, and KF.^50^, ^51^, ^52^ In contrast, urine 1-palmitoyl-2-oleoyl-GPC (16:0/18:1), also always selected, was linked to both KF and CKE in our earlier work.^21^ While serum levels of this metabolite have been associated with eGFR and proteinuria,^50^^,^^52^ no associations for its urine counterpart had been described before, classifying it as a novel finding in our previous project (see Table 2 of Steinbrenner et al^21^). Observations on both metabolites support their potential relevance for predicting risk of adverse kidney-related outcomes. Still, further studies to assess the biological meaning in the context of CKD progression are required.

Overall, the multimetabolite models showed good discrimination and low prediction error, with performance measures highest at year 2 and declining at more distant time points, a pattern also observed for the benchmark models (Fig 2). The observed decline in predictive value for more distant time points may stem from several factors. One explanation is that metabolite levels can vary over time due to biological variability, aging, or changes in lifestyle, thereby limiting the long-term predictive utility of baseline measurements alone.^53^ Supporting this, Lacruz et al^54^ showed that intraindividual changes in metabolite levels over time were themselves linked to all-cause mortality, suggesting the potential value of longitudinal measurements.

As with other proposed prognostic models, our findings reiterate that the risk prediction of adverse kidney-related outcomes can be improved in principle. However, the added predictive value beyond established tools like the KFRE remains small, limiting clinical utility.^14^^,^^15^^,^^45^^,^^55^^,^^56^ Future work should focus on integrating the different published results together with additional, novel data (eg, longitudinal measurements). Given the complex nature of CKD progression, a model may need to incorporate more extensive information from diverse data sources to achieve superior predictive performance. Ultimately, there is a trade-off between the added predictive value of a more complex model and the additional costs associated with increased model complexity, an aspect that needs to be addressed in a cost-benefit analysis.

One of the strengths of this study lies in the use of a well-characterized cohort with moderately decreased kidney function at baseline, comprehensive phenotyping, and long-term follow-up. Internal validation was performed using resampling techniques to assess the robustness of the findings and to approximate a lower bound for the predictive performance in external data.^38^^,^^57^ The CoxBoost method, chosen for developing the multimetabolite models, is particularly well-suited for high-dimensional time-to-event data, such as ours. Nonetheless, we cannot rule out the possibility that alternative modeling strategies might have yielded prognostic models with better performance.

Limitations of this project include the reliance on semiquantitative measurements from samples collected at a single time point (baseline) and the lack of external validation. Another important consideration is the quality of the biological samples: the samples used for metabolite profiling were collected at the baseline visit, stored at −80 °C, and only analyzed several years later. It was reported that such long-term storage, even at −80 °C, can affect metabolite levels.^58^^,^^59^

In summary, while we demonstrated that metabolites have predictive value for adverse kidney-related outcomes, their contribution to improving predictive performance appears to be of limited clinical relevance. Still, our findings on the predictive value of metabolites may also shed insights on metabolic pathways and processes related to the progression of CKD, thereby complementing existing knowledge and may even boost research on specific metabolites or pathways. Importantly, the development of a clinically applicable, improved risk equation for adverse kidney outcomes will require not only the integration of multiple suggestions on novel prognostic factor models but also the incorporation of additional data sources, such as longitudinal metabolite measurements, to reflect the complex nature of CKD progression.

## Supplementary Materials

Supplementary (PDF) /Figures S1-S10; Items S1-S10; *26-0115_SupplM.pdf*

Supplementary Excel File / Table S1-S18
