# Supplementary material for: Systematic Evaluation of Plasma and Urine Metabolites to Predict the Risk of Adverse Kidney-related Outcomes in Chronic Kidney Disease: The GCKD Study∗
Source: Kidney Med. 2026 May 13;8(7):101409. doi: 10.1016/j.xkme.2026.101409 (PMC13293684; doi:10.1016/j.xkme.2026.101409)
Supplement: Supplementary (PDF) — /Figures S1-S10; Items S1-S10; 26-0115_SupplM.pdf [file mmc1.pdf]

Systematic evaluation of plasma and urine metabolites to predict the risk of adverse kidney-related outcomes in chronic kidney disease: The GCKD study.

Elena Butz, Inga Steinbrenner, Ulla T. Schultheiss, Charlotte Behning, Harald Binder, Helena Hansmann, Wolfram Gronwald, Peter J. Oefner, Elke Schaeffner, Kai-Uwe Eckardt, Anna Köttgen, Peggy Sekula, on behalf of the GCKD Investigators

Contents

SUPPLEMENTARY MATERIAL 3
Item S1: List of GCKD participating institutions and investigators 3
Item S2: List and definitions of baseline variables in the GCKD study 4
Item S3: Urine and plasma metabolite measurements in the GCKD study 5
Item S4: Preprocessing of metabolite measurements for statistical analysis 6
Item S5: Evaluation of imputation quality 7
Item S6: Settings considered in the analysis to develop multi-metabolite models 9
Item S7: Parameter settings used for the development of multi-metabolite models using CoxBoost 10
Item S8: Parameter settings for the evaluation of the predictive performance of models 11
Item S9: Wrapper function to revise CoxBoost objects 13
Item S10: Stability of the metabolite selection with CoxBoost 15
SUPPLEMENTARY FIGURES 17
Figure S1: Workflow 17
Figure S2: Cumulative incidence functions of kidney failure and composite kidney endpoint 18
Figure S3: Change in AUC values and Brier scores when a single metabolite is added to a model predicting kidney failure 19
Figure S4: Change in AUC values and Brier scores when a single metabolite is added to a model predicting composite kidney endpoint 20
Figure S5: Illustration of the boosting algorithm for the development of a multi-metabolite model for kidney failure utilizing named plasma metabolites 21
Figure S6: Overview of metabolites selected in all models predicting composite kidney endpoint with either only named or all metabolites eligible 22
Figure S7: Apparent predictive performance of multi-metabolite models and respective benchmark models predicting kidney failure 23
Figure S8: Predictive performance of multi-metabolite models compared to respective benchmark models predicting kidney failure 24
Figure S9: Apparent net reclassification index and integrated discrimination improvement of six main models predicting kidney failure 25
Figure S10: Predictive performance of models developed on metabolites from either plasma, urine, or both 26

SUPPLEMENTARY TABLES 27
REFERENCES 28

SUPPLEMENTARY MATERIAL

Item S1: List of GCKD participating institutions and investigators

The nine study centers of the German Chronic Kidney Disease (GCKD) study in Germany are: RWTH Aachen University, Aachen; Charité – University-Medicine, Berlin; Friedrich-Alexander University, Erlangen; Albert-Ludwigs-University, Freiburg; Friedrich-Schiller University, Jena; Hannover Medical School, Hannover; Medical Faculty, Ruprecht-Karls University, Heidelberg; Medical Faculty, Ludwig-Maximilians-University, Munich; and Julius-Maximilians-University, Würzburg.

A list of nephrologists currently collaborating with the GCKD study is available at http://www.gckd.org.

- **University of Erlangen-Nürnberg**
  Kai-Uwe Eckardt, Heike Meiselbach, Markus P. Schneider, Mario Schiffer, Hans-Ulrich Prokosch, Barbara Bärthlein, Andreas Beck, André Reis, Arif B. Ekici, Susanne Becker, Ulrike Alberth-Schmidt, Anke Weigel, Sabine Marschall, Eugenia Schefler
- **University of Freiburg**
  Gerd Walz, Anna Köttgen, Ulla T. Schultheiß, Fruzsina Kotsis, Simone Meder, Erna Mitsch, Ursula Reinhard
- **RWTH Aachen University**
  Jürgen Floege, Turgay Saritas, Alice Groß
- **Charité, University Medicine Berlin**
  Elke Schaeffner, Seema Baid-Agrawal, Kerstin Theisen
- **Hannover Medical School**
  Hermann Haller
- **University of Heidelberg**
  Martin Zeier, Claudia Sommerer, Mehtap Aykac
- **University of Jena**
  Gunter Wolf, Martin Busch, Rainer Paul
- **Ludwig-Maximilians University of München**
  Thomas Sitter
- **University of Würzburg**
  Christoph Wanner, Vera Krane, Antje Börner-Klein, Britta Bauer
- **Medical University of Innsbruck, Division of Genetic Epidemiology**
  Florian Kronenberg, Julia Raschenberger, Barbara Kollerits, Lukas Forer, Sebastian Schönherr, Hansi Weissensteiner
- **University of Regensburg, Institute of Functional Genomics**
  Peter J. Oefner, Wolfram Gronwald
- **Institute of Medical Biometry, Informatics and Epidemiology, Medical Faculty, University of Bonn**
  Matthias Schmid, Jennifer Nadal

Item S2: List and definitions of baseline variables in the GCKD study

Variable	Comment	Missing values (N=5,217)
**Clinical information used in both prediction models MET+ and MET++ (KFRE factors)**		
Age	in years	0 (0%)
Sex	self-reported; male (0), female (1)	0 (0%)
eGFR	estimated glomerular filtration rate; in mL/min/1.73m²; eGFR was estimated using the Chronic Kidney Disease Epidemiology Collaboration (CKD-EPI) formula;[1] an IDMS traceable enzymatic assay (Creatinine Plus, Roche) was used to measure serum creatinine (mg/dL).	55 (1%)
UACR	urinary albumin-to-creatinine ratio; in mg/g; UACR was based on urinary creatinine (IDMS traceable enzymatic assay, Creatinine Plus, Roche; mg/dL) and urinary albumin (ALBU-XS assay (Roche/Hitachi Diagnostics GmbH, Mannheim, Germany; mg/L); for statistical analysis, UACR was ln-transformed (natural logarithm).	90 (2%)
**Further clinical information used in prediction model MET++**		
smoking	self-reported; non-smoker (0), ex-smoker (1), smoker (2); individuals reporting current daily or occasional smoking were classified as smokers.	16 (0%)
BMI	body mass index; in kg/m²; BMI was calculated from individual's body weight and height, and corrected for any amputations.	54 (1%)
BP, systolic	blood pressure, systolic; in mmHg; systolic BP was calculated as the mean out of three measurements spaced one minute apart after five minutes resting using a standardized device (OMRON M5 Professional, Mannheim, Germany).	34 (1%)
serum albumin	in g/L; serum albumin was quantified using a turbidimetric method (Tina-quant, Roche, Germany).	57 (1%)
CRP	high-sensitivity C-reactive protein; in mg/L; CRP was measured using an immunoturbidimetric test (CRPHS, Roche, Germany) on a Roche/Hitachi MODULAR (P); for statistical analysis, CRP was ln-transformed (natural logarithm).	56 (1%)
total cholesterol	in mg/dL; total cholesterol was measured using an enzymatic colorimetric method (CHOD-PAP, Roche, Germany) on a Roche/Hitachi MODULAR (P).	62 (1%)
diabetes	presence of diabetes at baseline; no (0) or yes (1); diabetes was defined as either a baseline serum hemoglobin A1c (HbA1c) of ≥6.5% or documented use of diabetes medication (Anatomical Therapeutic Chemical [ATC] code beginning with 'A10';[2]).	0 (0%)
CHD	prevalent coronary heart disease; no (0) or yes (1); CHD was defined as self-reported history of myocardial infarction, bypass operation, or percutaneous coronary intervention.[3]	2 (0%)
stroke	self-reported history of stroke; no (0) or yes (1)	2 (0%)
loop diuretics	self-reported intake of blood pressure-lowering medications: loop diuretics; no (0) or yes (1); group of loop diuretics was defined based on ATC codes (ATC 'C03C').	0 (0%)

Abbreviations: KFRE, kidney failure risk equation; **MET+**, metabolite model including major prognostic variables for kidney failure; **MET++,** metabolite model including major prognostic variables for kidney failure and further reported prognostic variables of adverse kidney-related outcomes

Item S3: Urine and plasma metabolite measurements in the GCKD study

Spot urine and plasma samples collected at baseline from participants of the German Chronic Kidney Disease (GCKD) study were used to determine metabolite levels using non-targeted liquid chromatography-mass spectrometry (LC-MS) analysis provided by Metabolon, Inc. (Durham, NC, USA)[4,5]. While plasma samples were measured in one batch (2020), urine samples had been analysed in three batches (2016-2017).

Sample preparation, LC-MS, and metabolite identification were described previously[6,7]. Briefly, four different UPLC-MS/MS methods were used for maximum coverage of hydrophilic and hydrophobic molecules by positive and negative ion mode electrospray ionization (range: 70-1000 *m/z* [mass-to-charge ratio])[8]. To identify metabolites, ion features in the GCKD study samples were compared to a reference library using an in-house software[9,10]. The Metabolon's reference library contains information on molecular mass, retention time, preferred adducts, in-source fragments and associated MS spectra of various chemical standards. It is continuously curated. Levels of the metabolites were measured as area under the integrals of respective peaks[10]. Raw area counts were normalized to account for inter-day instrument variation by the median value for each run-day and represent semi-quantitative (unit-less) measurements.

To align metabolite identification between assessed urine and plasma samples from the GCKD study, metabolite identification in processed urine samples was repeated at the same time plasma sample results were processed.

While some identified metabolites belong to different super-pathways (e.g., amino acids, xenobiotics), a large proportion of metabolites is still only partially characterized or unnamed (i.e., of unknown structural identity). Unless a named metabolite is marked by an asterisk, named metabolites conform to highest confidence level (level 1) of the Metabolomics Standards Initiative[11,12].

Item S4: Preprocessing of metabolite measurements for statistical analysis

After transfer of measurements to the Institute of Genetic Epidemiology (Medical Center – University of Freiburg, Germany), an in-house pipeline was used for data quality control and preparation of data for statistical analysis.

Details on quality control checks can be found elsewhere[6,7]. The quality-controlled datasets contain semi-quantitative measurements of 1,513 urine metabolites in 5,088 of 5,217 GCKD study participants (97.5%) and 1,416 plasma metabolites in 5,144 of 5,217 GCKD study participants (98.6%). Semi-quantitative levels show different degrees of completeness (range: 0-100%).

In preparation of the statistical analysis, urine measurements were normalized to harmonize differences in dilution using the probabilistic quotient method[13]. As in previous studies[6,14,15], all metabolite measurements were $\log_2$-transformed to reduce skewness in data.

Furthermore, non-xenobiotics with <50% missing levels and xenobiotics with <5% missing levels were imputed per matrix. Using *k*-nearest neighbor (*knn*) algorithm with k=10, the levels of 1,096 plasma metabolites and 1,129 urine metabolites were imputed[16]. Annotation of imputed plasma and urine metabolites are listed in **Supplementary Tables 2** and **3**.

Item S5: Evaluation of imputation quality

To assess whether the imputation of missing measurements affects analyses, two sensitivity analyses were conducted:

1. **Question:** does the imputation affect the correlation structure of metabolite pairs within the same matrix?

For this purpose, Spearman correlation coefficients of each metabolite pair of metabolites per matrix were calculated from unimputed and imputed data. Respective coefficients for a given pair are plotted against each other in the **figure**:

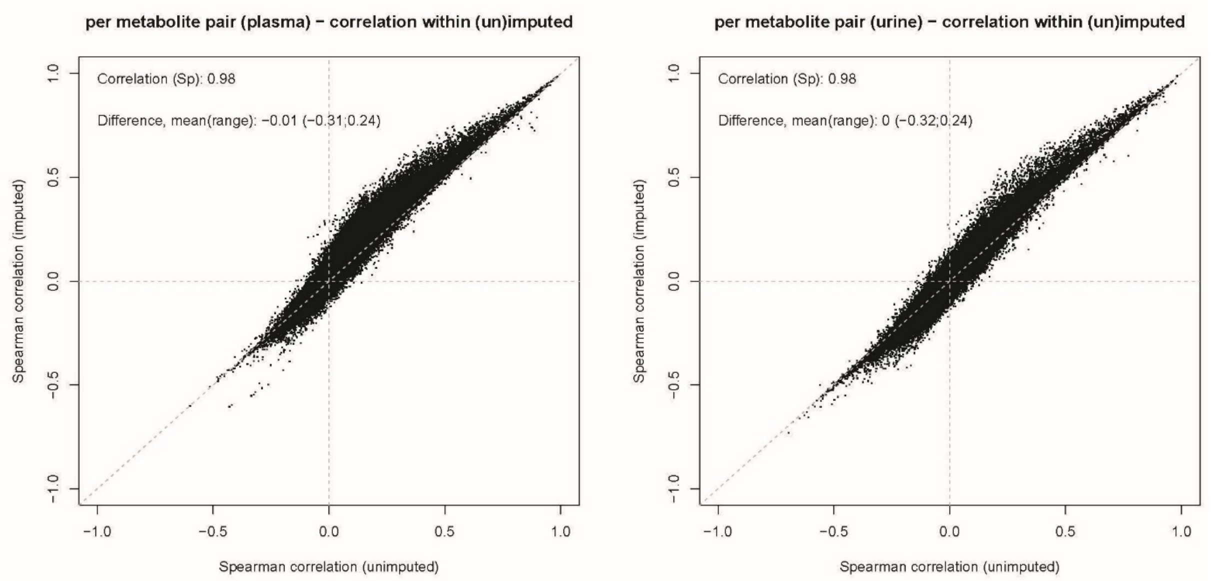

For each pair of metabolites within each matrix, the Spearman correlation coefficient was calculated with unimputed (x-axis) and imputed (y-axis) data and displayed against each other. left: for plasma metabolites, right: for urine metabolites.

Conclusion: Imputation of missing values essentially retains correlation structure.

2. **Question:** does the imputation affect the direction and significance of associations?

For this purpose, the association analysis with adjustment for age, sex, eGFR and ln-transformed UACR as reported in Steinbrenner et al. was repeated using the imputed data[17]. The table shows Spearman correlation coefficients for adjusted effect estimates (p-values) based on the results from each stage and setting:

Matrix		Plasma		Urine	
**Outcome**		**KF**	**CKE**	**KF**	**CKE**
**Analysis stage**	**Discovery**	0.98 (0.95)	0.99 (0.96)	0.97 (0.92)	0.98 (0.94)
	**Replication**	0.98 (0.95)	0.98 (0.95)	0.96 (0.91)	0.98 (0.94)
	**Meta-analysis**	0.99 (0.95)	0.99 (0.96)	0.97 (0.93)	0.98 (0.94)

For illustration, the scatter plots display obtained effect estimates (beta) and -log10 p-values obtained from the meta-analyses:

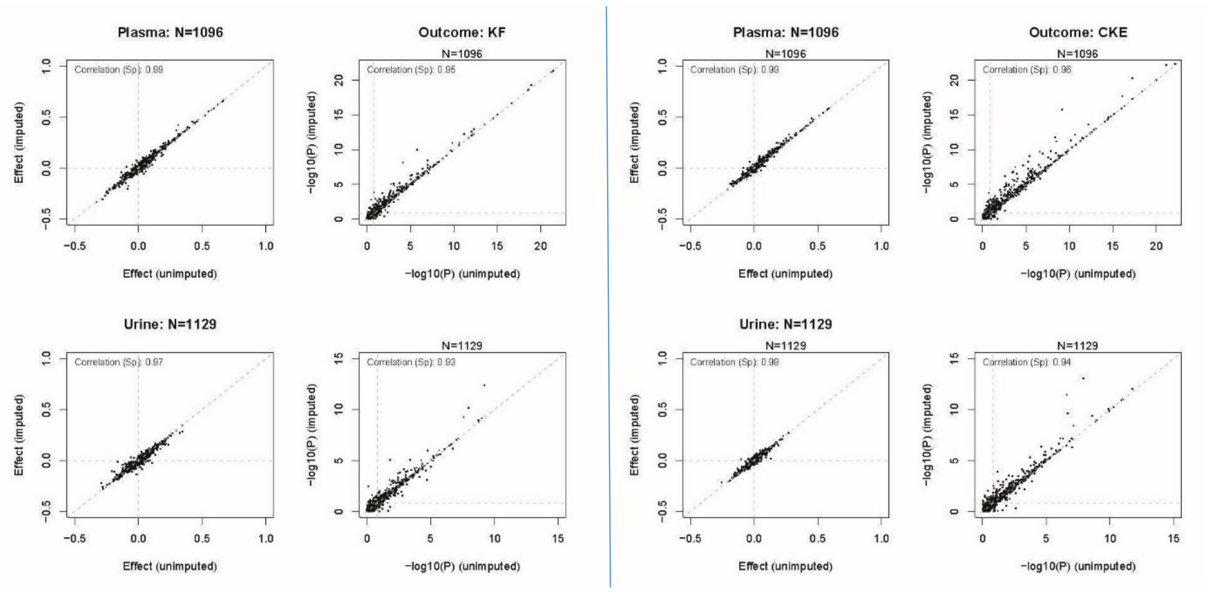

Left: results for kidney failure (KF), right: results for of composite kidney endpoint (CKE), upper row: analysis of plasma metabolites, lower row: analysis of urine metabolites.

Abbreviations: Sp, Spearman correlation coefficient.

Conclusion: Overall, results obtained from the analysis of imputed data show good agreement with those of unimputed data.

Item S6: Settings considered in the analysis to develop multi-metabolite models

To systematically assess the potential of plasma and urinary metabolites, three general settings for the development of multi-metabolite prediction models were considered:

Setting No	Benchmark model		(Multi-)metabolite model	
1	**Bench**	*Null model*	**MET**	Bench model + metabolite(s)
2	**Bench+**	Including four KFRE variables	**MET+**	Bench+ model + metabolite(s)
3	**Bench++**	Including an extended set of prognostic variables	**MET++**	Bench++ model + metabolite(s)

The four KFRE variables comprise age, sex, eGFR, and UACR (ln-transformed) were included. Details of the variables included in the extended set of prognostic factors which also comprises the four KFRE variables, can be found in **Item S2**.

Abbreviations: KFRE, Kidney Failure Risk Equation.

In the main analysis, the primary outcome kidney failure was considered. Multi-metabolite models were derived using both plasma and urine metabolites that were named by Metabolon.

Additional analyses:

- The same approach was used considering also metabolites unnamed by Metabolon in the derivation of multi-metabolite models to assess the predictive value of unnamed metabolites.
- Moreover, analyses were similarly conducted using either plasma metabolites only or urine metabolites only to assess whether predictive of each matrix on its own.

Finally, the same procedure was used for the secondary outcome, the composite kidney outcome.

Item S7: Parameter settings used for the development of multi-metabolite models using CoxBoost

For the development of multi-metabolite models, penalized regression was utilized using the component-wise boosting algorithm *CoxBoost* (v1.5, https://github.com/binderh/CoxBoost)[18]. A 2-step approach was used (i) to determine the number of necessary boosting steps for a given dataset and (ii) to conduct feature selection with the determined number of steps.

List of chosen parameters:

1. Determination of number of necessary boosting steps using the function *cv.CoxBoost*

   input:
   - vector with time to event/censoring information
   - vector with event indicator
   - matrix with exposure information

   parameters:
   - maxstepno = 500 (in stability assessment: 750)
   - K = 10
   - type = ”verweij”
   - penalty = 9*number of events of interest
   - *for models with clinical information*: unpen.index = list of numbers indicating columns in exposure matrix containing clinical information

2. Selection of metabolites using the function *CoxBoost*

   input: *as above*

   parameters:
   - stepno = number of necessary boosting steps determined in step 1
   - penalty = 9*number of events of interest (default)
   - criterion = "score"
   - cmprsk = "sh"
   - standardize = FALSE
   - *for models with clinical information*: unpen.index = list of numbers indicating columns in exposure matrix containing clinical information

Item S8: Parameter settings for the evaluation of the predictive performance of models

To comprehensively assess the predictive performance of each model, we used the R package *riskRegression* (version 2023.01.19; https://github.com/tagteam/riskRegression) developed for regression models with survival outcomes in the presence of competing risks.[19,20]

In the following, template code is provided for the evaluation of model's predictive performance:

data:	data frame
time:	variable of observation time from study entry until event of interest, competing event or censoring, whatever occurred first (in days)
event:	variable indicating the type of event (0: censored, 1: event of interest, 2: competing event)
model*x*:	CoxBoost object

(A) initialization of standard parameters

```
tpl <- 365.25 * c(2,4,5,6)       # time points of interest
b <- 100                         # number boosting steps
tlist <- sort(unique(dat[dat[,<time>] <=6 * 365.25, <time>]))
                                 # list of all time points in the data
```

(B) evaluation of apparent performance

AUC and Brier score

```
riskRegression::Score(list(<label1>=<model1>, <label2>=<model2>, …),
                      formula=Hist(<time>, <event>) ~ 1, data = <data>,
                      se.fit = TRUE, times = tpl, metrics = c("auc","brier"),
                      cause = 1)
```

Integrated Brier score

```
riskRegression::Score(list(<label1>=<model1>, <label2>=<model2>, …),
                      formula=Hist(<time>, <event>) ~ 1, data = <data>,
                      times = tlist, summary = "ibs",
                      metrics = "brier")
```

(C) evaluation with bootstrap crossvalidation

```
set.seed(<number>)
riskRegression::Score(list(<label1>=<model1>, <label2>=<model2>, …),
                      formula=Hist(<time>, <event>) ~ 1, data = <data>,
                      se.fit=TRUE, times = tpl, metrics=c("auc","brier"),
                      B = b, split.method = "bootcv")
```

If more than one model is stated in the call of the ***Score*** function, the function automatically delivers contrasts between all pairs of models in the list.

Item S9: Wrapper function to revise CoxBoost objects

The following code was used to call CoxBoost within a wrapper function providing a revised CoxBoost object that can then be forwarded to riskRegression:

```
#' Formula interface for function \code{CoxBoost} of package \code{CoxBoost}.
#'
#' Formula interface for function \code{CoxBoost} of package \code{CoxBoost}.
#'
#' See \code{CoxBoost}.
#' @aliases coxboost
#' @param formula An event-history formula for competing risks of the
#' form \code{Hist(time,status)~sex+age} where \code{status} defines
#' competing events and right censored data. The code for right
#' censored can be controlled with argument \code{cens.code}, see man
#' page the function \code{\link{Hist}}.
#' @param data A data.frame in which the variables of formula are
#' defined.
#' @param cv If \code{TRUE} perform cross-validation to optimize the
#' parameter \code{stepno}. This calls the function \code{cv.CoxBoost}
#' whose arguments are prefix controlled, that is \code{cv.K=7} sets
#' the argument \code{K} of \code{cv.CoxBoost} to \code{7}.  If
#' \code{FALSE} use \code{stepno}.
#' @param cause The cause of interest in competing risk models.
#' @param penalty See \code{CoxBoost}.
#' @param ... Arguments passed to either \code{CoxBoost} via
#' \code{CoxBoost.arg} or to \code{cv.CoxBoost} via
#' \code{cv.CoxBoost.arg}.
#' @return See \code{CoxBoost}.
#' @author Thomas Alexander Gerds \email{tag@@biostat.ku.dk}
#' @seealso See \code{CoxBoost}.
#' @references See \code{CoxBoost}.
#' @keywords survival
#' @export
coxboost                                                                   <-
function(formula,data,cv=TRUE,cause=1,penalty,seed,unpen.index=NA,cmprsk="",cri
terion="",standardize=TRUE,stepno=100,...){
  call <- match.call(expand.dots=TRUE)
  formula.names <- try(all.names(formula),silent=TRUE)
  if (!(formula.names[2]=="Hist")) stop("The left hand side of formula should
look like this: Hist(time,event).")
  actual.terms <- terms(formula,data=data)
  formula <- eval(call$formula)
  response <- model.response(model.frame(formula,data))
  Time <- as.numeric(response[,"time"])
  if (attr(response,"model")=="competing.risks"){
    ## adapt the event variable
    Event <- rep(2,NROW(response))
    thisCause <- as.numeric(response[,"event"]==cause)
    Event[thisCause==1] <- 1
    Status <- as.numeric(response[,"status"])
    Event[Status==0] <- 0
  }
  else{
    ## survival
    Event <- as.numeric(response[,"status"])
  }
  X    <-    model.matrix(actual.terms,data=data)[,-c(1),drop=FALSE]##    remove
intercept
  if (NCOL(X)<=1) stop("CoxBoost needs at least two covariates.")
  if (missing(penalty)) penalty <- sum(Event==1)*(9)
  cv.defaults=list(maxstepno=200,K=10,penalty=penalty)
  CoxBoost.defaults=list(stepno=100,penalty=penalty)
```

```
  args <- prodlim::SmartControl(call= list(...),
                        keys=c("cv","CoxBoost"),
                        ignore=c("formula","data","cv","cause"),

forced=list("cv"=list(time=Time,status=Event,x=X),"CoxBoost"=list(time=Time,sta
tus=Event,x=X)),

defaults=list("cv"=cv.defaults,"CoxBoost"=CoxBoost.defaults),
                        ignore.case=FALSE,
                        replaceDefaults=FALSE,
                        verbose=TRUE)
  if (cv==TRUE){
    cv.step <- do.call("cv.CoxBoost",args$cv)
    args$CoxBoost$stepno <- cv.step$optimal.step
  } else {
    args$CoxBoost$stepno <- stepno
  }
  set.seed(seed)
  if (!is.na(unpen.index[[1]])) args$CoxBoost$unpen.index <- unpen.index
  if (cmprsk!="")               args$CoxBoost$cmprsk      <- cmprsk
  if (criterion!="")            args$CoxBoost$criterion   <- criterion
  if (standardize!=TRUE)        args$CoxBoost$standardize <- standardize
  args$CoxBoost$seed = NULL
  cb <- do.call("CoxBoost",args$CoxBoost)
  out <- list(coxboost=cb,
              stepno=args$CoxBoost$stepno,
              call=call,
              formula=formula,
              response=response,
              cb.coef=cb$coefficients[length(cb$coefficients[,1]),])
  class(out) <- "coxboost"
  out
}


##' @export
predictRisk.coxboost <- function(object,newdata,times,cause,...){
    if (missing(cause)) stop("missing cause")
    newcova <- model.matrix(terms(object$formula,data=newdata),

data=model.frame(object$formula,data=newdata,na.action=na.fail))[,-c(1)]
    newcova <- newcova[,object$coxboost$xnames]
    if (attr(object$response,"model")!="competing.risks"){
        p <- predict(object$coxboost,newcova,type="risk",times=times)
    } else{
        p <- predict(object$coxboost,newdata=newcova,type="CIF",times=times)
    }
    if (is.null(dim(p))) {
        if (length(p)!=length(times))
            stop("Prediction failed (wrong number of times)")
    } else{
        if (NROW(p) != NROW(newdata) || NCOL(p) != length(times))
            stop(paste("\nPrediction  matrix  has  wrong  dimension:\nRequested
newdata  x  times:  ",NROW(newdata),"  x  ",length(times),"\nProvided  prediction
matrix: ",NROW(p)," x ",NCOL(p),"\n\n",sep=""))
    }
    p
}
```

Item S10: Stability of the metabolite selection with CoxBoost

To study the stability of the selection of metabolites by CoxBoost, we generated 100 random samples without replacement with 63.2% of the individuals from each data set used in the analysis.[21] The selection of metabolites using CoxBoost was then conducted by repeating the entire procedure, including the determination of the required boosting steps. Names of metabolites and how often they had been selected across the 100 random samples were extracted.

Results

As these resampled datasets were smaller and included fewer events, the median number of boosting steps and selected metabolites was correspondingly lower (**Table S11**).

Metabolites included in the original model were selected far more frequently in the resampling procedure than those not originally selected:

Figure: Inclusion frequencies of metabolites according to their selection in the original model

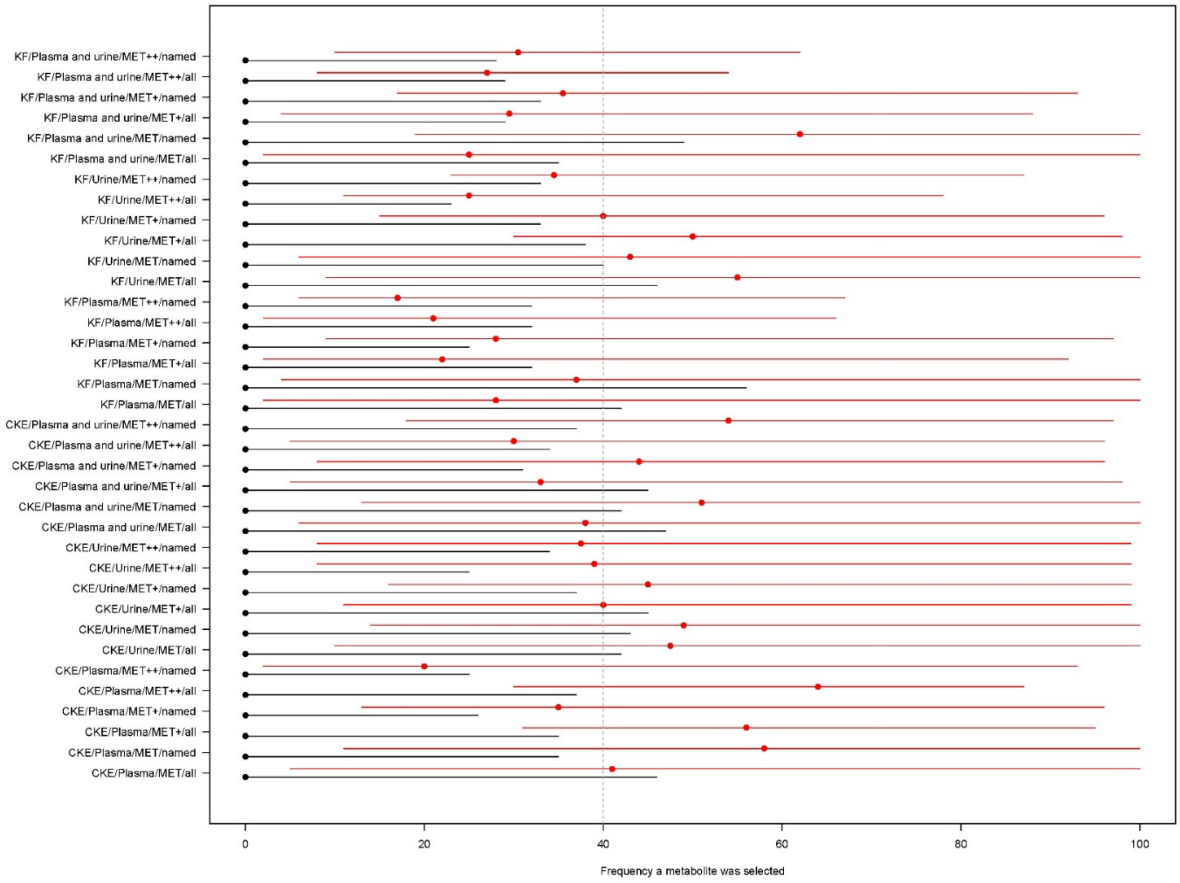

For each of 36 settings in total (**Item S6**), the distribution of metabolite selection frequency across 100 bootstrap samples is shown (point: median, line: minimum and maximum). Metabolites were categorized into a group of metabolites that were selected originally in the respective setting (red) and that were not selected originally (black). The grey vertical line represents a threshold at 40%.

Fewer than half of the metabolites not selected in the main model appeared in any bootstrap sample, with the maximum frequency being 56 out of 100 subsamples (**Table S11**).

In contrast, metabolites that had been selected in the original model were often reselected, with individual inclusion frequencies reaching up to 100% in MET models and up to 99% in the other models.

The proportion of metabolites from the main analysis that were selected in at least 40 of the 100 subsamples ranged from 15% to 89% (median 46.5%).

Conclusion

Metabolites that were selected into the model originally, were also selected more often in bootstrap samples.

SUPPLEMENTARY FIGURES

Figure S1: Workflow

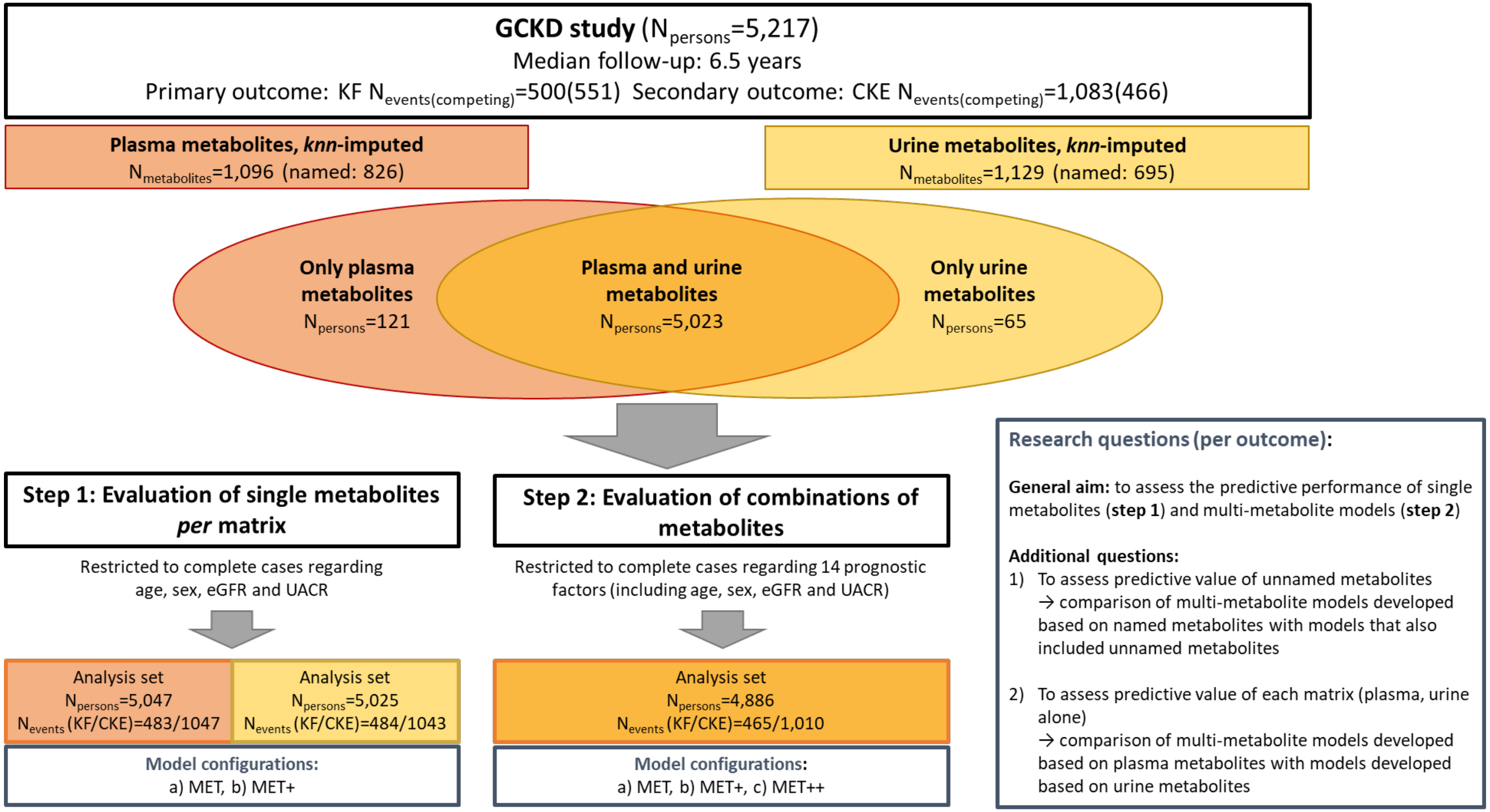

See **Item S3** and **Item S4** for details on measurements of metabolites and their preparations for the analyses.

See **Item S6** for details on model settings of MET, MET+ and MET++ and included clinical factors.

Abbreviations: **CKE,** composite kidney endpoint; **eGFR,** estimated glomerular filtration rate; **KF,** kidney failure; **UACR,** urinary albumin-to-creatinine ratio.

In total, plasma measurements were available for 5,144 persons and urine for 5,088 (overlap N=5,023). For a total of 8 persons no measurements were available at all.

Figure S2: Cumulative incidence functions of kidney failure and composite kidney endpoint

(A) outcome: kidney failure (primary endpoint)

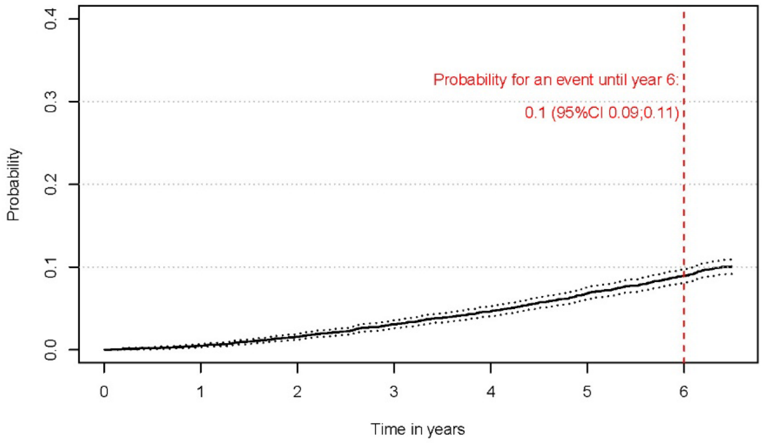

Numbers at risk:

Year 0	Year 1	Year 2	Year 3	Year 4	Year 5	Year 6
4886	4752	4561	4371	4150	3898	3591

(B) outcome: composite kidney endpoint (secondary endpoint)

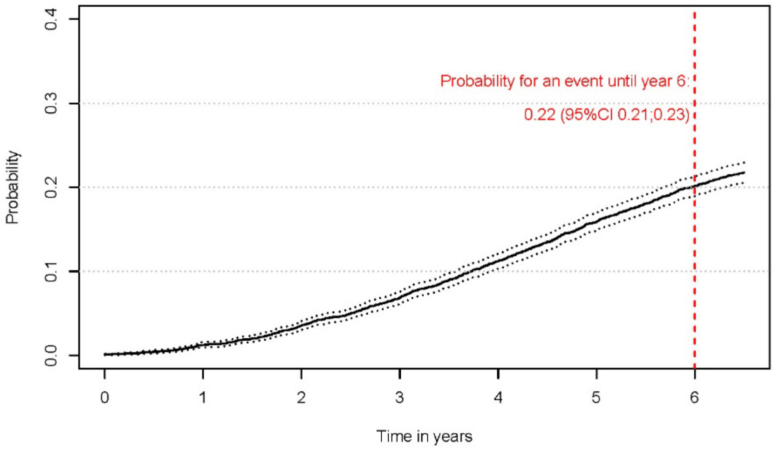

Numbers at risk:

Year 0	Year 1	Year 2	Year 3	Year 4	Year 5	Year 6
4886	4718	4473	4208	3871	3522	3159

To account for competing risks, the cumulative incidence function in the main analysis set (N=4,884) was estimated to extract the probability for an event until year six.

Figure S3: Change in AUC values and Brier scores when a single metabolite is added to a model predicting kidney failure

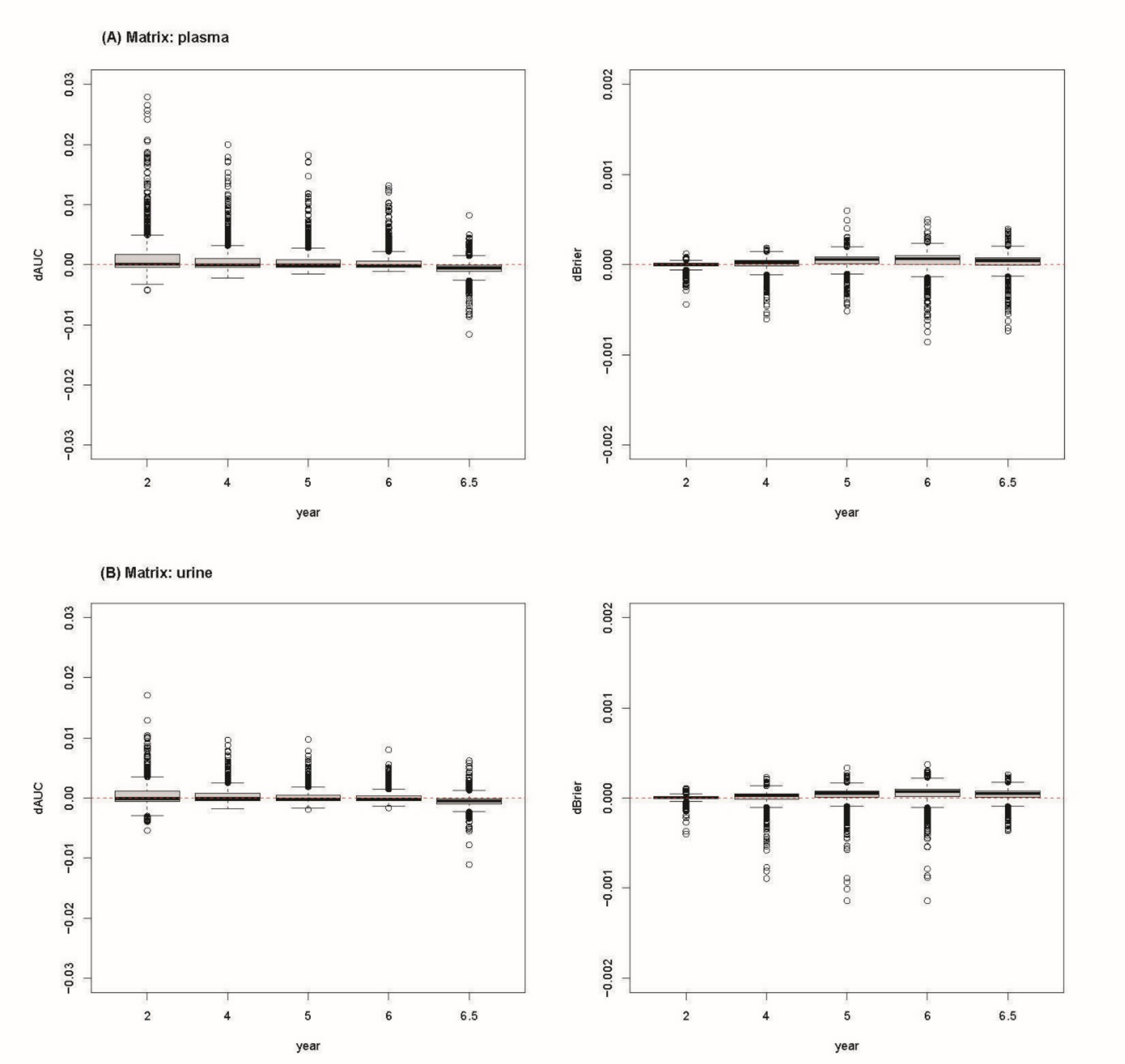

Change in AUC (dAUC) and Brier score (dBrier) is measured as the difference between the AUC or Brier score from the model with four KFRE variables (age, sex, eGFR, and ln-transformed UACR) and the model that additionally includes a single metabolite. Positive values of dAUC reflect improvement when adding the metabolite. For Brier score, negative values reflect improvement. The plot shows the box plots for dAUC (left) and dBrier (right) at specific times per matrix.

Conclusion: While metabolites do not improve the prediction of the respective outcome on average, there are several instances where metabolites can improve the prediction (dAUC >0 or dBrier <0), visible as upward outliers in AUC difference plots and downward outliers in Brier score difference plots.

Abbreviations: AUC, area under the receiver operating characteristic curve; **KFRE,** kidney failure risk equation; **eGFR,** estimated glomerular filtration rate; **UACR,** urinary albumin-to-creatinine ratio.

Figure S4: Change in AUC values and Brier scores when a single metabolite is added to a model predicting composite kidney endpoint

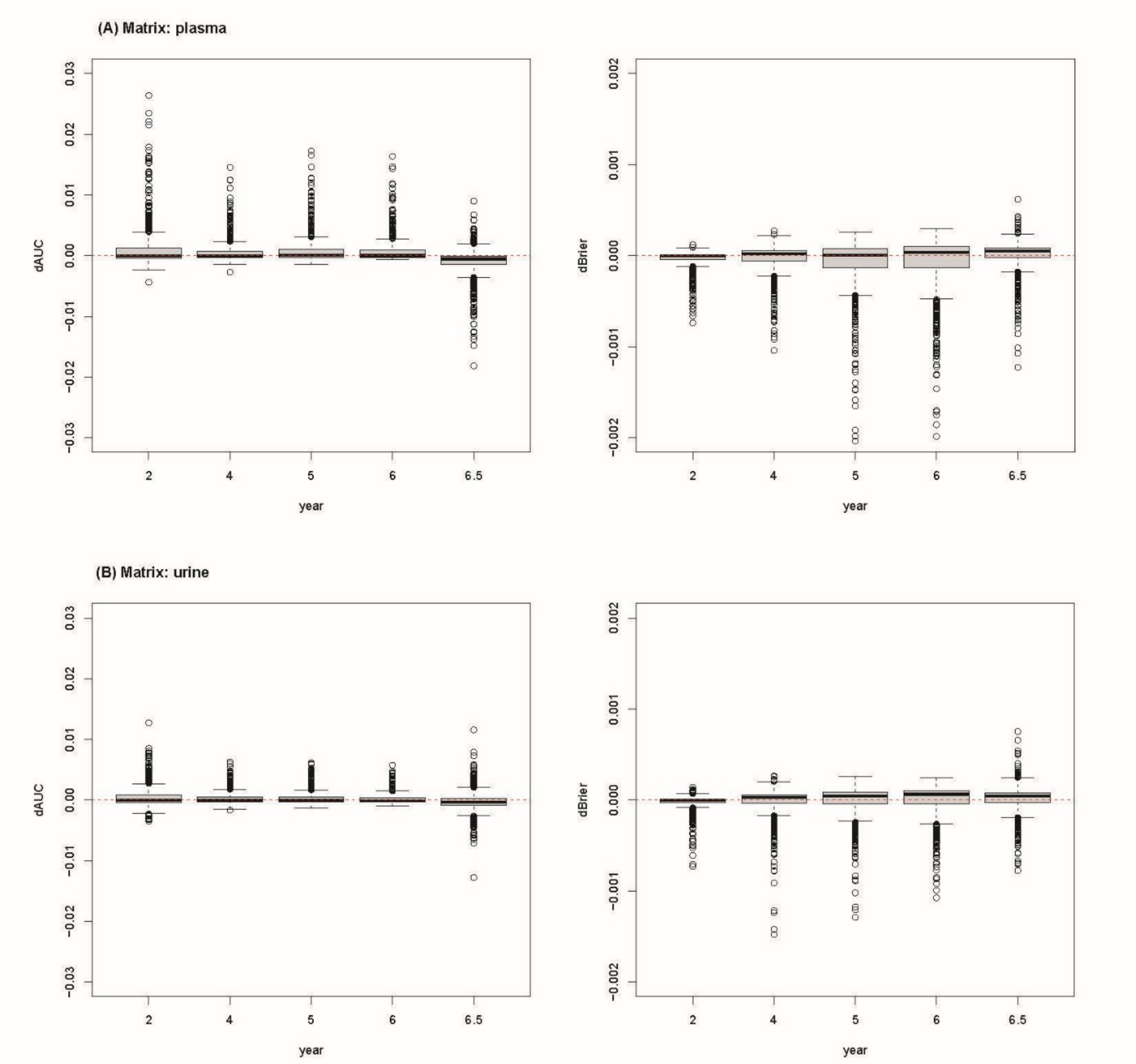

Change in AUC (dAUC) and Brier score (dBrier) is measured as the difference between the AUC or Brier score from the model with four KFRE variables (age, sex, eGFR, and ln-transformed UACR) and the model that additionally includes a single metabolite. Positive values of dAUC reflect improvement when adding the metabolite. For Brier score, negative values reflect improvement. The plot shows the box plots for dAUC (left) and dBrier (right) at specific times per matrix.

Conclusion: While metabolites do not improve the prediction of the respective outcome on average, there are several instances where metabolites can improve the prediction (dAUC >0 or dBrier <0).

Abbreviations: AUC, area under the receiver operating characteristic curve; **KFRE,** kidney failure risk equation; **eGFR,** estimated glomerular filtration rate; **UACR,** urinary albumin-to-creatinine ratio.

Figure S5: Illustration of the boosting algorithm for the development of a multi-metabolite model for kidney failure utilizing named plasma metabolites

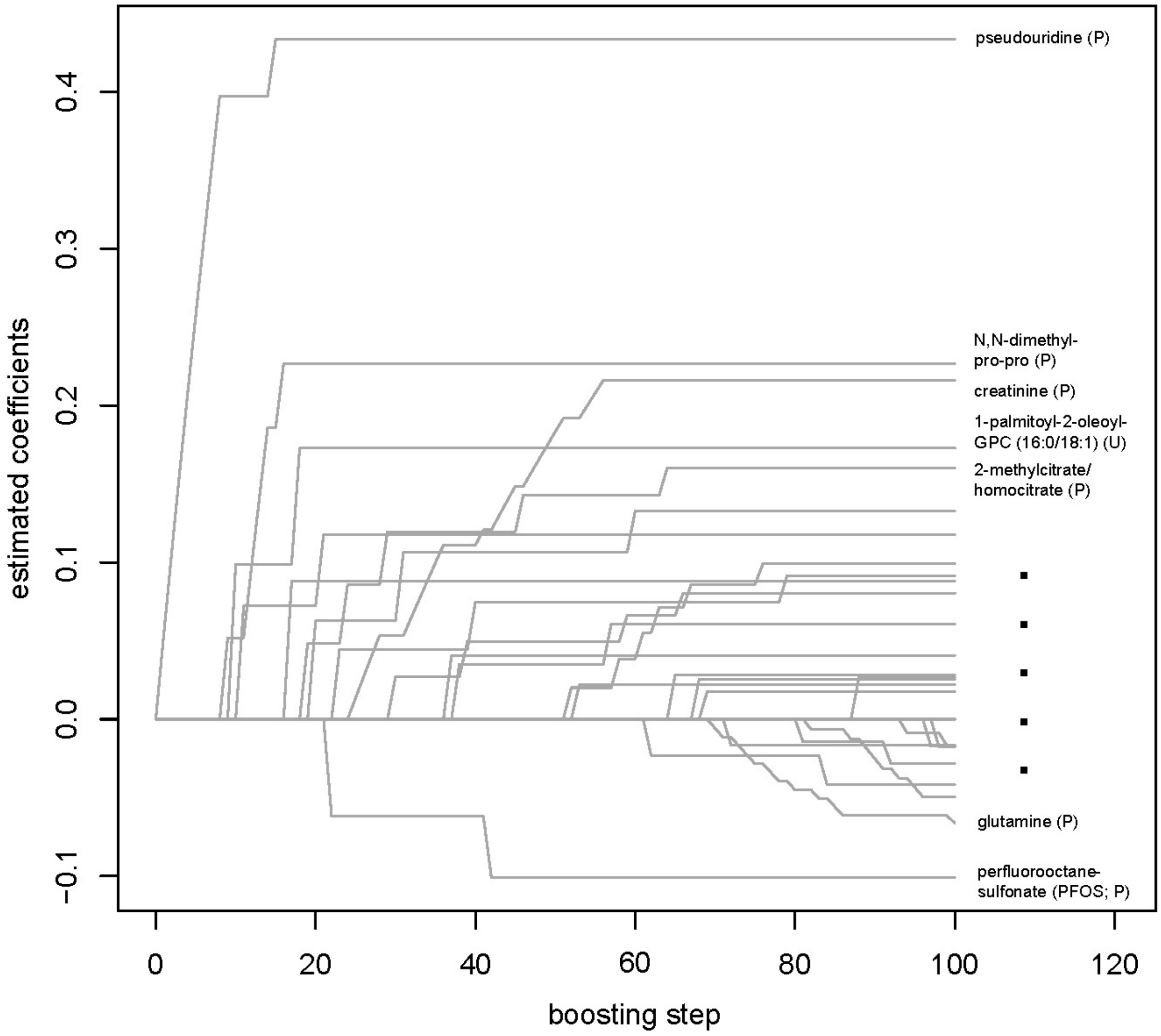

For the development of multi-metabolite models, penalized regression was utilized using the component-wise boosting algorithm CoxBoost (v1.5, https://github.com/binderh/CoxBoost)[18]. In a first step, the optimal number of boosting steps is determined using 10-fold cross-validation. In the here presented setting (outcome: kidney failure, model: metabolites only [MET], matrix: named plasma and urine metabolites), the determined number of boosting steps was 100. In a second step, the selection of metabolites was done using the predefined number of steps. At each step, the coefficient of one variable is updated and any metabolite with a non-zero coefficient is selected into the model. This figure shows the coefficient paths for this specific setting. Selected paths are annotated with the names of the respective metabolite. In brackets, P stands for plasma and U for urine. In the end, 27 metabolites had been seleted.

See **Item S7** for details on parameters used in the call of CoxBoost and **Table S10** for all selected metabolites.

Figure S6: Overview of metabolites selected in all models predicting composite kidney endpoint with either only named or all metabolites eligible

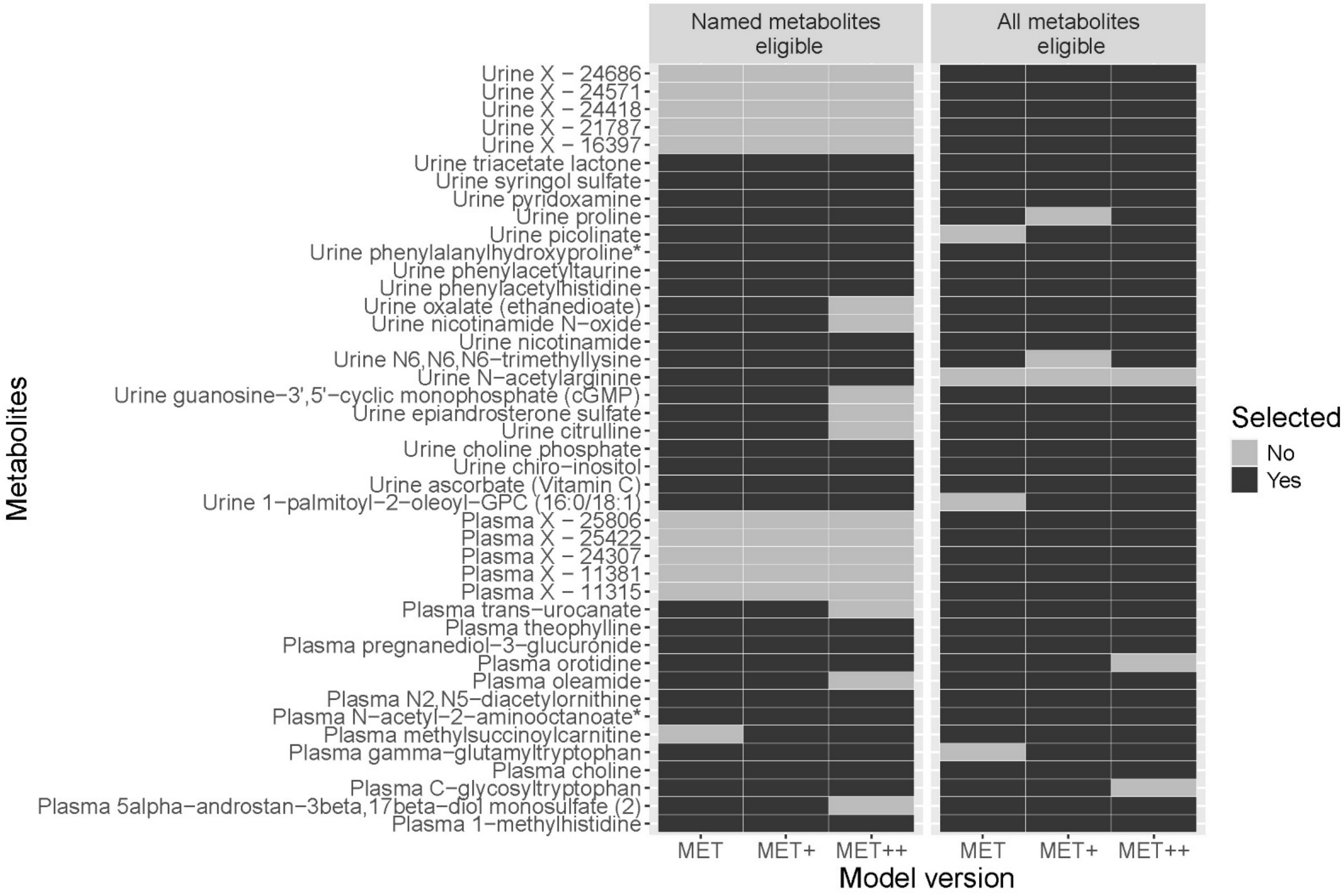

Multi-metabolite models were derived under three different settings (**Item S6**), with either only named metabolites eligible or all metabolites eligible. This heatmap highlights metabolites that were chosen in all three settings of either set of eligible metabolites.
Model settings:
MET: model incorporating only metabolite information
MET+: metabolite model including metabolite information and the four variables from the Kidney Failure Risk Equation (age, sex, eGFR, and ln-transformed UACR)
MET++: metabolite model including metabolite information and an extended set of prognostic factors (see **Item S2** for details)
Abbreviations: eGFR, estimated glomerular filtration rate; **UACR,** urinary albumin-to-creatinine ratio.

Figure S7: Apparent predictive performance of multi-metabolite models and respective benchmark models predicting kidney failure

(A) Matrix: plasma and urine, named metabolites

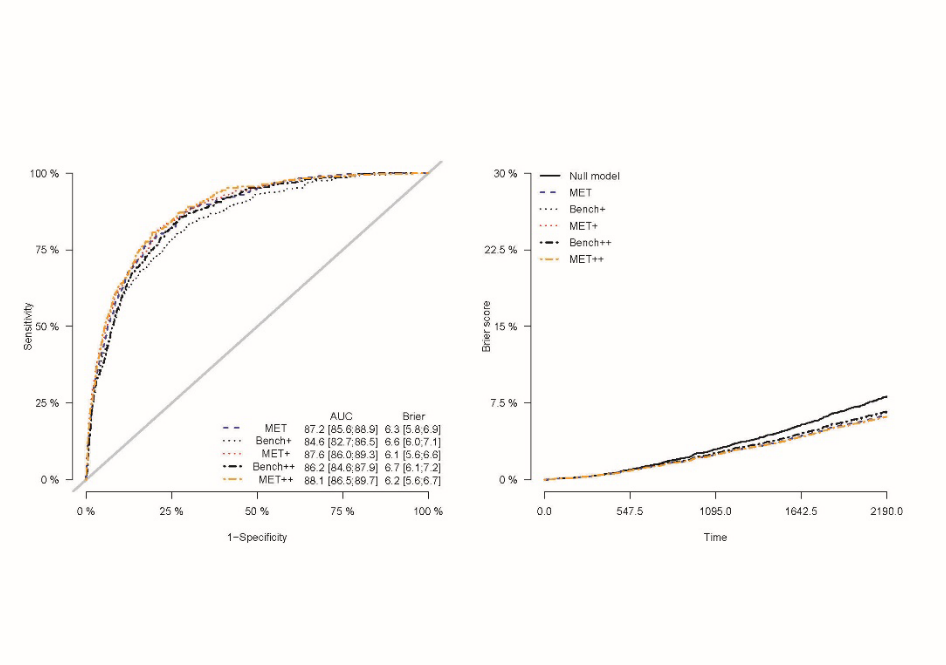

(B) Matrix: plasma and urine, all metabolites

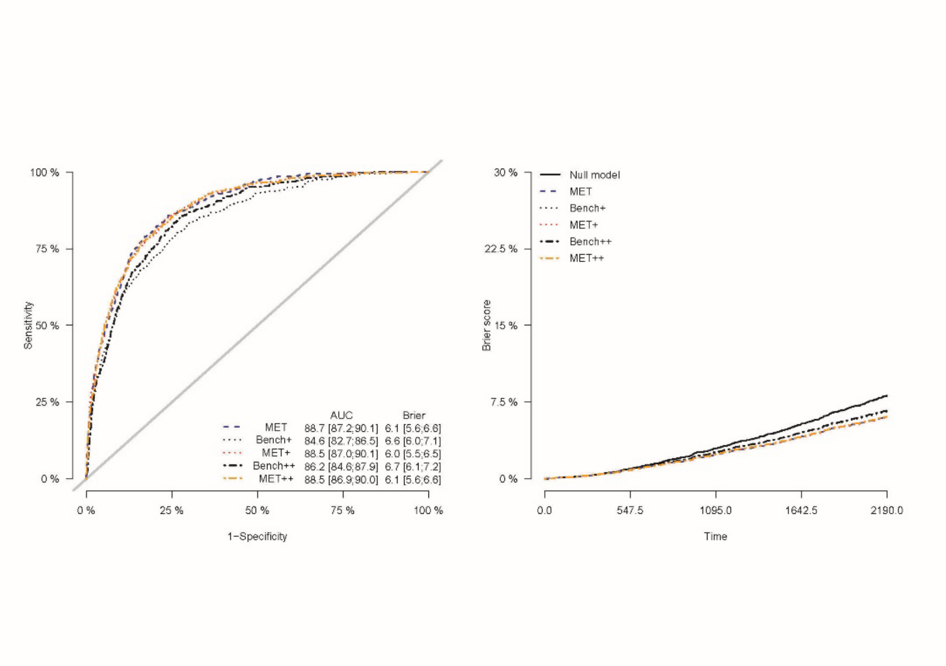

Left: receiver operating characteristics curve at time = 6 years; Right: Prediction error curve
Null model: model without any explanatory variable; **MET:** metabolite model**; Bench+:** model only including major prognostic variables for kidney failure; **MET+**: metabolite model including major prognostic variables for kidney failure; **Bench++:** model only including major prognostic variables for kidney failure and further reported prognostic variables of adverse kidney-related outcomes; **MET++**: metabolite model including major prognostic variables for kidney failure and further reported prognostic variables of adverse kidney-related outcomes; See **Item S2** for details on prognostic variables included.

Figure S8: Predictive performance of multi-metabolite models compared to respective benchmark models predicting kidney failure

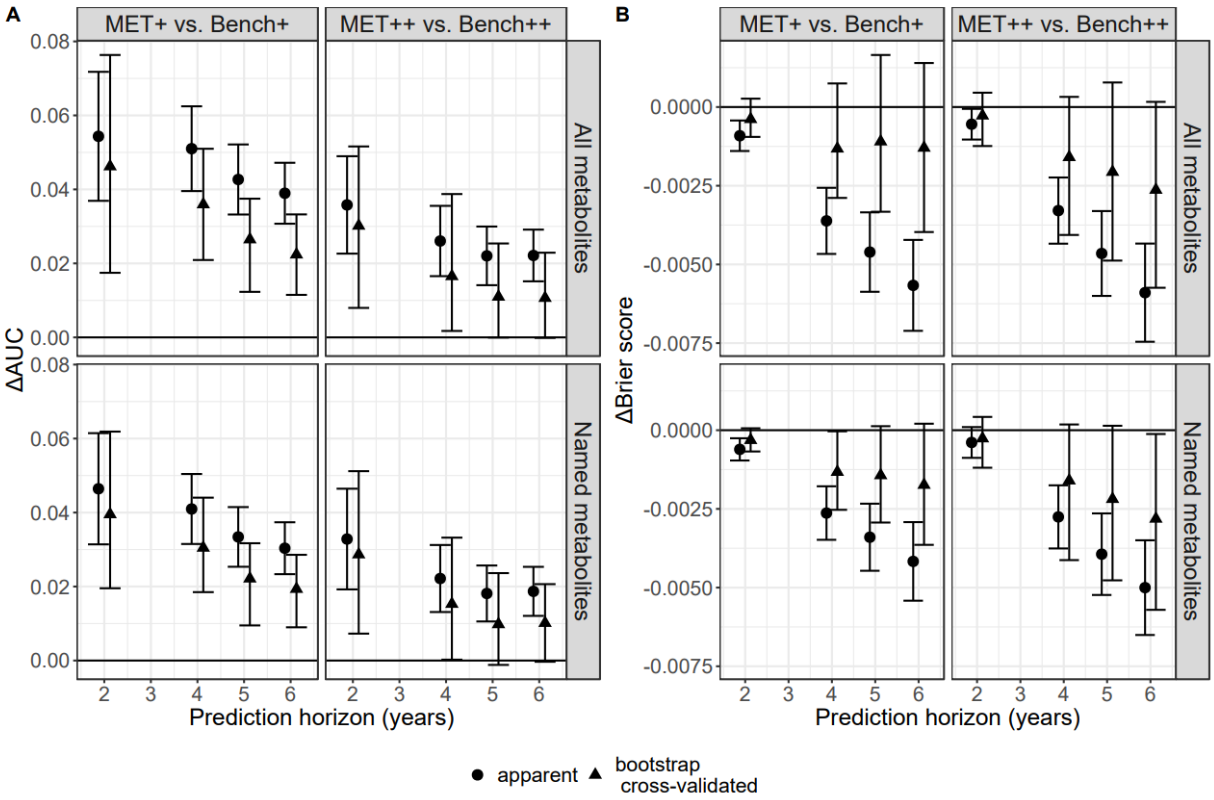

The differences in the area under the receiver operating characteristic curve (AUC, Panel A) and Brier score (Panel B) between multi-metabolite models and their respective benchmark models. Multi-metabolite models significantly outperform benchmark models in terms of AUC for the closer time points, but not for more distant time points and not in terms of the (cross-validated) Brier score. **MET:** metabolite model**; Bench+:** model only including major prognostic variables for kidney failure; **MET+**: metabolite model including major prognostic variables for kidney failure; **Bench++:** model only including major prognostic variables for kidney failure and further reported prognostic variables of adverse kidney-related outcomes; **MET++**: metabolite model including major prognostic variables for kidney failure and further reported prognostic variables of adverse kidney-related outcomes; See **Item S2** for details on prognostic variables included.

Figure S9: Apparent net reclassification index and integrated discrimination improvement of six main models predicting kidney failure

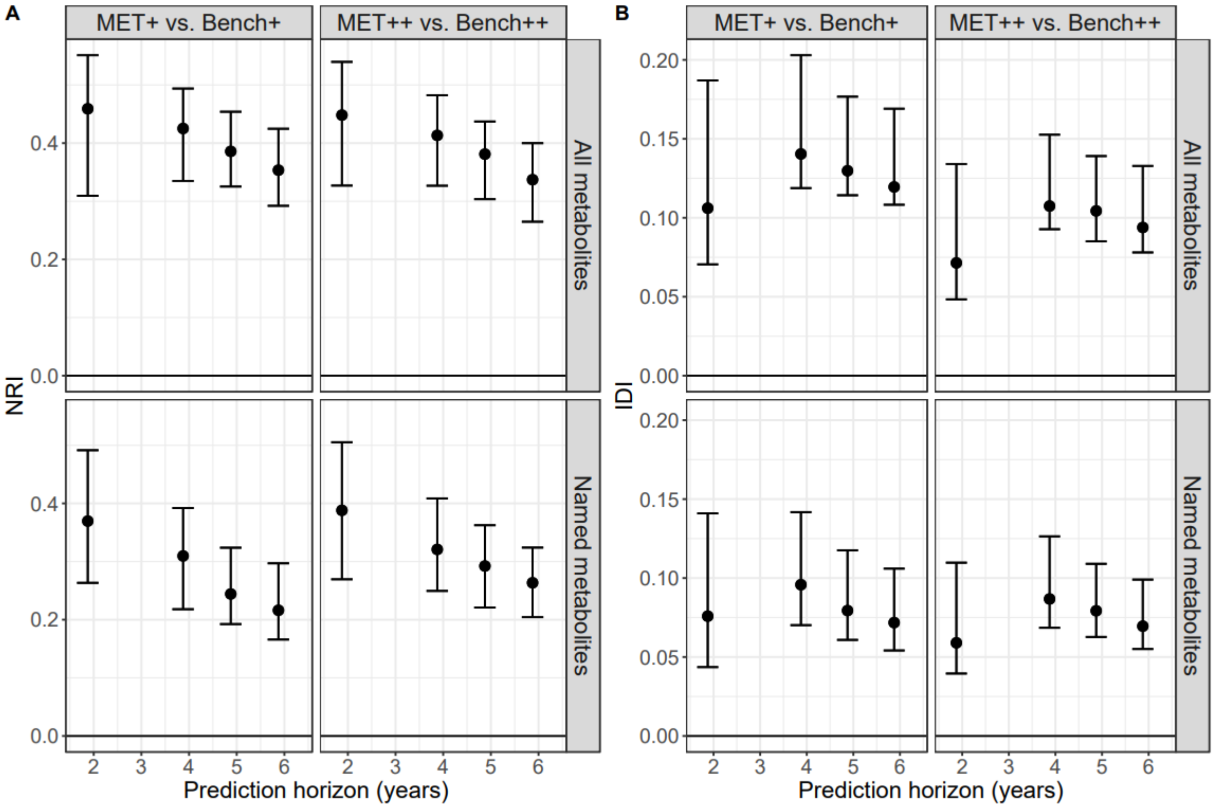

Both the net reclassification index (NRI, panel **A**) and integrated discrimination improvement (IDI, panel **B**) indicate that models with metabolites perform significantly better than their respective benchmark models, especially if not only named but all metabolites could be chosen to be included in the model. **MET:** metabolite model**; Bench+:** model only including major prognostic variables for kidney failure; **MET+**: metabolite model including major prognostic variables for kidney failure; **Bench++:** model only including major prognostic variables for kidney failure and further reported prognostic variables of adverse kidney-related outcomes; **MET++**: metabolite model including major prognostic variables for kidney failure and further reported prognostic variables of adverse kidney-related outcomes; See **Item S2** for details on prognostic variables included. Numerical results are presented in **Table S15**.

Figure S10: Predictive performance of models developed on metabolites from either plasma, urine, or both

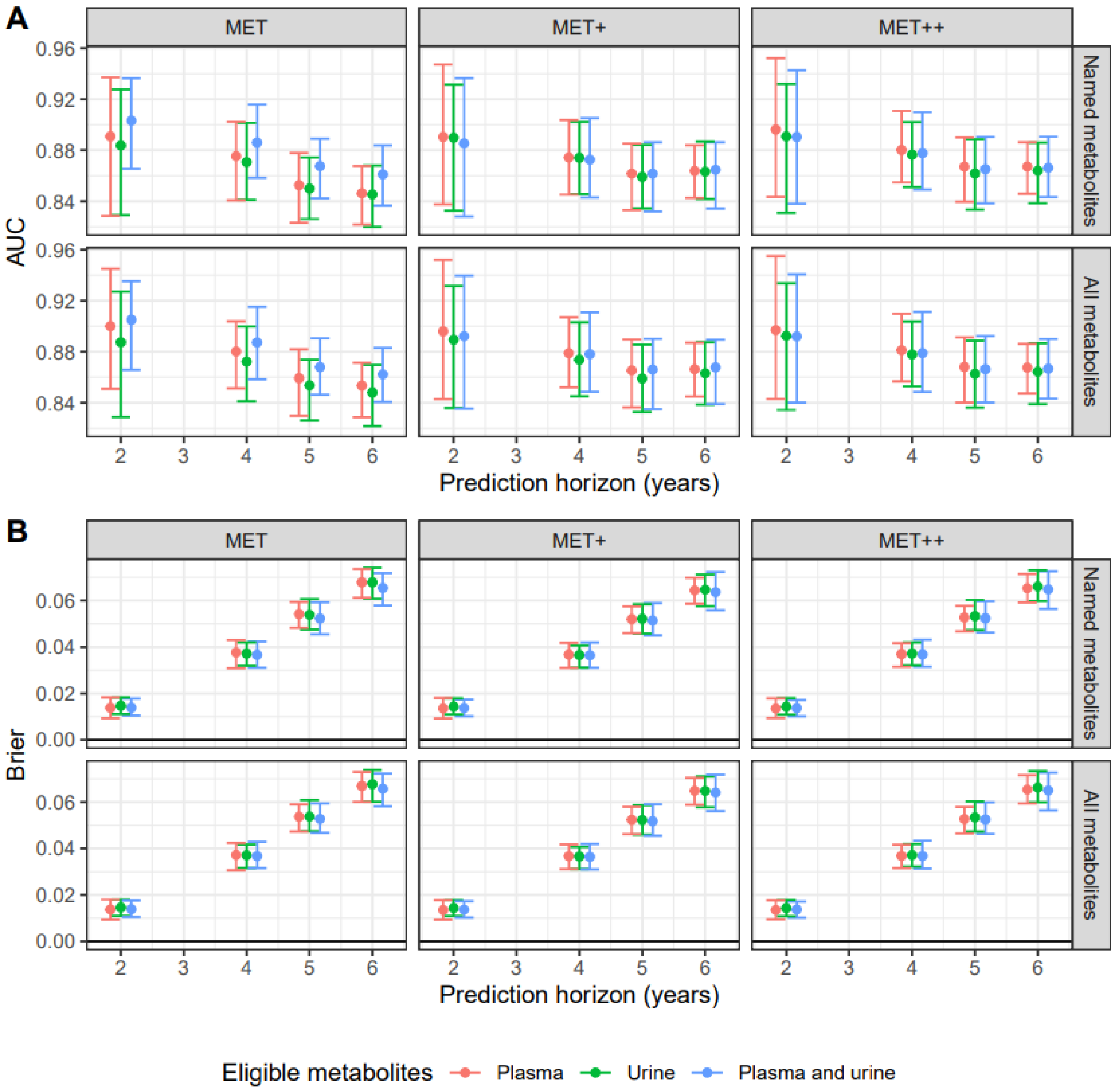

Predictive performance of models developed on metabolites from either plasma, urine, or both was very similar. MET: metabolite model**; Bench+:** model only including major prognostic variables for kidney failure; **MET+**: metabolite model including major prognostic variables for kidney failure; **Bench++:** model only including major prognostic variables for kidney failure and further reported prognostic variables of adverse kidney-related outcomes; **MET++**: metabolite model including major prognostic variables for kidney failure and further reported prognostic variables of adverse kidney-related outcomes; See **Item S2** for details on prognostic variables included

SUPPLEMENTARY TABLES

See separate excel file

REFERENCES

1. Levey AS, Stevens LA, Schmid CH, et al. A new equation to estimate glomerular filtration rate. *Ann Intern Med*. 2009;150(9):604-612. doi:10.7326/0003-4819-150-9-200905050-00006

2. Schmidt IM, Hübner S, Nadal J, et al. Patterns of medication use and the burden of polypharmacy in patients with chronic kidney disease: the German Chronic Kidney Disease study. *Clin Kidney J*. 2019;12(5):663-672. doi:10.1093/ckj/sfz046

3. Titze S, Schmid M, Köttgen A, et al. Disease burden and risk profile in referred patients with moderate chronic kidney disease: composition of the German Chronic Kidney Disease (GCKD) cohort. *Nephrol Dial Transplant*. 2015;30(3):441-451. doi:10.1093/ndt/gfu294

4. Eckardt KU, Bärthlein B, Baid-Agrawal S, et al. The German Chronic Kidney Disease (GCKD) study: design and methods. *Nephrol Dial Transplant*. 2012;27(4):1454-1460. doi:10.1093/ndt/gfr456

5. Prokosch HU, Mate S, Christoph J, et al. Designing and implementing a biobanking IT framework for multiple research scenarios. *Stud Health Technol Inform*. 2012;180:559-563.

6. Schlosser P, Li Y, Sekula P, et al. Genetic studies of urinary metabolites illuminate mechanisms of detoxification and excretion in humans. *Nat Genet*. 2020;52(2):167-176. doi:10.1038/s41588-019-0567-8

7. Sekula P, Tin A, Schultheiss UT, et al. Urine 6-Bromotryptophan: Associations with Genetic Variants and Incident End-Stage Kidney Disease. *Sci Rep*. 2020;10(1):10018. doi:10.1038/s41598-020-66334-w

8. Shin SY, Fauman EB, Petersen AK, et al. An atlas of genetic influences on human blood metabolites. *Nat Genet*. 2014;46(6):543-550. doi:10.1038/ng.2982

9. Dehaven CD, Evans AM, Dai H, Lawton KA. Organization of GC/MS and LC/MS metabolomics data into chemical libraries. *J Cheminformatics*. 2010;2(1):9. doi:10.1186/1758-2946-2-9

10. Jump RLP, Polinkovsky A, Hurless K, et al. Metabolomics analysis identifies intestinal microbiota-derived biomarkers of colonization resistance in clindamycin-treated mice. *PloS One*. 2014;9(7):e101267. doi:10.1371/journal.pone.0101267

11. Sumner LW, Amberg A, Barrett D, et al. Proposed minimum reporting standards for chemical analysis Chemical Analysis Working Group (CAWG) Metabolomics Standards Initiative (MSI). *Metabolomics Off J Metabolomic Soc*. 2007;3(3):211-221. doi:10.1007/s11306-007-0082-2

12. Schrimpe-Rutledge AC, Codreanu SG, Sherrod SD, McLean JA. Untargeted Metabolomics Strategies-Challenges and Emerging Directions. *J Am Soc Mass Spectrom*. 2016;27(12):1897-1905. doi:10.1007/s13361-016-1469-y

13. Dieterle F, Ross A, Schlotterbeck G, Senn H. Probabilistic quotient normalization as robust method to account for dilution of complex biological mixtures. Application in 1H NMR metabonomics. *Anal Chem*. 2006;78(13):4281-4290. doi:10.1021/ac051632c

14. Steinbrenner I, Schultheiss UT, Kotsis F, et al. Urine Metabolite Levels, Adverse Kidney Outcomes, and Mortality in CKD Patients: A Metabolome-wide Association Study. *Am J Kidney Dis Off J Natl Kidney Found*. 2021;78(5):669-677.e1. doi:10.1053/j.ajkd.2021.01.018

15. Schlosser P, Scherer N, Grundner-Culemann F, et al. Genetic studies of paired metabolomes reveal enzymatic and transport processes at the interface of plasma and urine. *Nat Genet*. 2023;55(6):995-1008. doi:10.1038/s41588-023-01409-8

16. Do KT, Wahl S, Raffler J, et al. Characterization of missing values in untargeted MS-based metabolomics data and evaluation of missing data handling strategies. *Metabolomics Off J Metabolomic Soc*. 2018;14(10):128. doi:10.1007/s11306-018-1420-2

17. Steinbrenner I, Schultheiss UT, Bächle H, et al. Associations of Urine and Plasma Metabolites With Kidney Failure and Death in a Chronic Kidney Disease Cohort. *Am J Kidney Dis Off J Natl Kidney Found*. 2024;84(4):469-481. doi:10.1053/j.ajkd.2024.03.028

18. Binder H, Allignol A, Schumacher M, Beyersmann J. Boosting for high-dimensional time-to-event data with competing risks. *Bioinformatics*. 2009;25(7):890-896. doi:10.1093/bioinformatics/btp088

19. Gerds TA, Kattan MW. *Medical Risk Prediction: With Ties to Machine Learning*. 1st ed. Chapman and Hall/CRC; 2021. doi:10.1201/9781138384484

20. Gerds TA, Ohlendorff JS, Ozenne B. riskRegression: Risk Regression Models and Prediction Scores for Survival Analysis with Competing Risks. Published online December 22, 2011:2023.12.21. doi:10.32614/CRAN.package.riskRegression

21. Binder H, Schumacher M. Adapting prediction error estimates for biased complexity selection in high-dimensional bootstrap samples. *Stat Appl Genet Mol Biol*. 2008;7(1):Article12. doi:10.2202/1544-6115.1346
